# Structure‐ and Ligand‐Based Discovery of Novel 3‐Chymotrypsin‐Like Protease Nonpeptidomimetic Hits

**DOI:** 10.1002/cmdc.202501083

**Published:** 2026-04-09

**Authors:** Sabrina Silva‐Mendonça, Donald Seanego, Christopher Jurisch, Melina Mottin, Flávia Nader Motta, Beatriz S. A. Rodrigues, Gilberto S. M. Junior, Alexandra Maria dos Santos Carvalho, Fábio Muniz de Oliveira, Sunniva Sigurdardóttir, Per Sunnerhagen, Izabela Marques Dourado Bastos, Richard Gessner, Kelly Chibale, Carolina Horta Andrade

**Affiliations:** ^1^ Laboratory of Molecular Modeling and Drug Design (LabMol) Faculdade de Farmácia Universidade Federal de Goiás Goiânia Brazil; ^2^ Center for the Research and Advancement in Fragments and Molecular Targets (CRAFT) School of Pharmaceutical Sciences at Ribeirão Preto University of São Paulo Ribeirão Preto Brazil; ^3^ Center for Excellence in One Health (CESU) Universidade Federal de Goiás Goiânia Brazil; ^4^ Holistic Drug Discovery and Development Center (H3D) University of Cape Town Rondebosch South Africa; ^5^ Laboratory of Pathogen–Host Interaction (LIPH) Institute of Biological Sciences Universidade de Brasília Brasília Brazil; ^6^ Faculdade de Ceilândia Universidade de Brasília Brasília Brazil; ^7^ McGowan Institute for Regenerative Medicine, Department of Surgery University of Pittsburgh Pennsylvania USA; ^8^ Department of Chemistry and Molecular Biology University of Gothenburg Gothenburg Sweden; ^9^ Center for Excellence in Artificial Intelligence (CEIA) Institute of Informatics Universidade Federal de Goiás Goiânia Brazil

**Keywords:** 3‐chymotrypsin‐like protease, antiviral agents, Computer‐Aided Drug Design, computational chemistry, drug discovery, M^pro^, machine learning

## Abstract

The SARS‐CoV‐2 3‐chymotrypsin‐like (3CL^pro^) protease is a key target for the development of COVID‐19 therapeutics. While ensitrelvir and nirmatrelvir are approved drugs for treatment, the continuous research and development for new antiviral drugs is necessary to combat the emergence of variants and other related viruses. This study employed structure‐ and ligand‐based computer‐assisted approaches to identify new 3CL^pro^ nonpeptidomimetic inhibitors. Using data from COVID Moonshot, NCATS, and the literature, computational methods such as shape‐based, ensemble docking, and machine learning (ML) techniques were developed, achieving robust validation metrics: AUC = 87%, EF = 7, BEDROC = 60% for shape‐based; AUC = 87%, EF = 7.03, BEDROC = 62% for ensemble docking, and ACC = 81%, MCC = 62% for ML models, combing Random forest + ECFP4 fingerprint. These models were utilized in virtual screening (VS) campaigns using the H3D and ChemBridge libraries, from which six promising hits with IC_50_ values ≤80 µM were identified, including LabMol‐499 with an IC_50_ of 13.71 µM and a *K*_i_ of 21.74 µM. Moreover, we found that LabMol‐499 acts as a noncompetitive, reversible inhibitor of 3CL^pro^. These findings provide a foundation for hit‐to‐lead optimization of new nonpeptidomimetic 3CL^pro^ inhibitors.

## Graphical abstract

A multistep virtual screening cascade, integrating shape‐based, ensemble docking, and quantitative structure–activity relationship (QSAR) models, was employed to screen 1.5 million compounds. This funnel identified a novel 3‐chymotrypsin‐like protease (3CL^pro^) inhibitor, LabMol‐499, which was experimentally validated using a Förster Resonance Energy Transfer (FRET) assay, revealing an IC_50_ of 13.71 µM and a *K*_i_ of 21.4 µM.

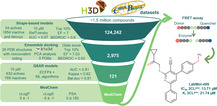

## 1 Introduction

The coronavirus disease 2019 (COVID‐19) pandemic, caused by severe acute respiratory syndrome coronavirus 2 (SARS‐CoV‐2), has precipitated a global health crisis with profound socioeconomic consequences [1]. Among the extensively investigated therapeutic targets, the SARS‐CoV‐2 3‐chymotrypsin‐like protease (3CL^pro^ or M^pro^) stands out due to its role in viral replication and its high degree of conservation across variants, making it an attractive candidate for broad‐spectrum antiviral development. Crucially, 3CL^pro^ exhibits a unique substrate specificity (Ala‐Val‐Leu‐Gln↓Ser‐Gly‐Phe‐Arg), which distinguishes it from human proteases and minimizes the risk of off‐target effects [2], thereby enhancing its therapeutic potential. The active site of SARS‐CoV‐2 3CL^pro^ is organized into several subsites (S1, S2, S3, S4, and S1′), each contributing to substrate recognition and inhibitor binding. The S1 subsite is mainly defined by residues His163, Glu166, and Phe140, which form a polar pocket that explicitly accommodates the conserved glutamine at the P1 position of substrates. The S2 subsite is a hydrophobic pocket primarily shaped by His41, Met49, and Met165, which favors the binding of bulky hydrophobic groups, such as leucine or phenylalanine, at position 2 (P2). The S3 subsite is relatively shallow and solvent‐exposed, composed of residues such as Met165 and Gln189, which allows for more structural flexibility for diverse chemical groups at P3. The S4 subsite is a small, hydrophobic cavity involving Gln189, Met165, and surrounding flexible loop residues, which can accommodate various substituents at P4. Finally, the S1′ subsite lies adjacent to the canonical active site Cys145‐His41 dyad and includes residues Gly143, Ser144, and Cys145, contributing to stabilization of the scissile peptide bond and playing a critical role in catalysis [3, 4, 5].

To date, the antiviral arsenal against COVID‐19 includes drugs such as remdesivir (an RNA‐dependent RNA polymerase inhibitor) [6], molnupiravir (a mutagenic ribonucleoside) [7], and nirmatrelvir/ritonavir (Paxlovid) [8], a 3CL^pro^ inhibitor combined with a pharmacokinetic enhancer, respectively. While these therapies have demonstrated clinical efficacy, emerging reports of resistance mutations [9, 10] and treatment limitations (e.g., ritonavir's drug‐drug interactions) [11] underscore the urgent need for novel inhibitors able to circumvent limitations associated with current drug regimens.

In this context, Computer‐Aided Drug Design (CADD) has emerged as an indispensable tool in accelerating drug discovery, particularly during global health emergencies [12, 13]. CADD methodologies, including molecular docking [14], pharmacophore modeling [14], and machine learning (ML)‐driven and generative modeling [15, 16], have been instrumental in identifying and optimizing inhibitors of SARS‐CoV‐2 targets, such as 3CL^pro^. For instance, CADD‐enabled approaches have successfully identified noncovalent and covalent inhibitors (e.g., ensitrelvir and nirmatrelvir), validating the protease as a druggable target [17, 18]. However, the continued evolution of SARS‐CoV‐2 necessitates the exploration of structurally diverse scaffolds to overcome potential resistance mechanisms.

Our study combines cutting‐edge computational and experimental strategies to identify new 3CL^pro^ inhibitors. By integrating advanced multimethod computational modeling, including ensemble docking, shape‐based screening, and ML‐based activity prediction, we screened extensive compound libraries to find the most promising hits. Our experimental validation, conducted with sensitive fluorescence resonance energy transfer (FRET) assays, precisely measures inhibitory activity and kinetics, providing deep insights into binding mechanisms. Moreover, validating these findings in yeast‐based cell models confirms their biological relevance. In alignment with recent perspectives highlighting the superior effectiveness of hybrid and multidisciplinary approaches in cheminformatics [19], our protocol employs a hierarchical consensus strategy. By integrating ligand‐centric (shape‐based), structure‐centric (ensemble docking), and data‐centric (ML) filters, this study mitigates the limitations inherent to individual methods. This integrative workflow enabled prioritizing high‐confidence candidates, resulting in a robust experimental hit rate and the identification of diverse non‐peptidomimetic scaffolds.

## 2 Results and Discussion

### 2.1 Data Compilation and Preparation

A comprehensive survey of scientific literature and publicly accessible databases—including ChEMBL V. 30 [20] (CHEMBL44955832 [21]), the NCATS COVID‐19 Open Data Portal [22], and the PostEra COVID Moonshot initiative database [23] was conducted to identify chemical entities with biological activity against 3CL^pro^. Compounds were compiled based on the availability of Simplified Molecular Input Line Entry System (SMILES) [24] and experimental bioactivity data (IC_50_ or *K*_i_) obtained exclusively via FRET assays to enhance experimental uniformity. Chemical structures were prepared according to the protocols of Fourches and colleagues [25, 26] (detailed in Supporting Information S1, Methods section).

Priority was given to IC_50_ values to maintain consistency; where duplicates existed, IC_50_ entries were retained. Following a rigorous curation workflow (standardization and removal of mixtures/salts), an initial dataset was evaluated for SAR integrity. As discussed in Section 3.3, a quantitative activity cliff (AC) analysis revealed significant heterogeneity in the literature‐derived subset (discontinuity score = 0.95), likely due to varying laboratory conditions. To ensure model reliability and minimize experimental artifacts, this subset was excluded from the final training set.

The final dataset comprised 1401 compounds. Applying a bioactivity threshold of 10 µM [14] resulted in a balanced distribution of 632 actives and 769 inactives (ratio ∼ 1:1.2). This natural balance precluded the need for synthetic oversampling or undersampling, allowing the ML models to be trained on the original experimental distribution (Figure S1).

For structure‐based validation (ensemble docking and shape‐based modeling), a subset of 54 active and 10 inactive noncovalent inhibitors was utilized. To rigorously test these models, 1944 decoys were generated from the ZINC13 database [27], maintaining a 1:36 active‐to‐decoy ratio. The decoy generation protocol matched physicochemical properties (e.g., molecular weight and rotatable bonds) to prevent artificial enrichment based on trivial volume discrepancies, ensuring that retrieval was driven by specific 3D shape and chemical feature similarity [28, 29].

Finally, a consensus strategy was employed: a noncovalent ensemble docking protocol served as a mechanistic filter to prioritize reversible 3CL^pro^ binders, followed by evaluation via ML models to identify compounds combining optimal binding geometry with potent physicochemical profiles.

### 2.2 Collection and Selection of the 3D Structures of 3CL^pro^

To overcome the limitations of rigid‐receptor docking, normal mode analysis (NMA) was applied to sample the conformational flexibility of the 3CL^pro^ binding site. NMA provides a computationally efficient approximation of the protein's functional motions by calculating the low‐frequency vibrational modes, which correspond to large‐scale domain movements often associated with ligand binding [30, 31]. By perturbing the crystal structure along these modes, we generated a diverse ensemble of receptor conformations, enabling the identification of cryptic pockets and accommodating a broader range of chemotypes during the virtual screening (VS) campaign.

The Protein Data Bank [32] (PDB) provided a curated dataset comprising 316 3D structures of SARS‐CoV‐2 3CL^pro^, available up to April 30, 2021. These structures were systematically categorized into three groups: apo (ligand‐free), holo (ligand‐bound), and fragment (see Supporting Information S3). From the holo subset, structures demonstrating allosteric ligand binding modes were excluded. Criteria such as resolution (≤2 Å) and the presence of noncovalent interactions with 3CL^pro^ were applied, resulting in a final selection of 14 high‐quality structures for further detailed analysis.

**FIGURE 1:**
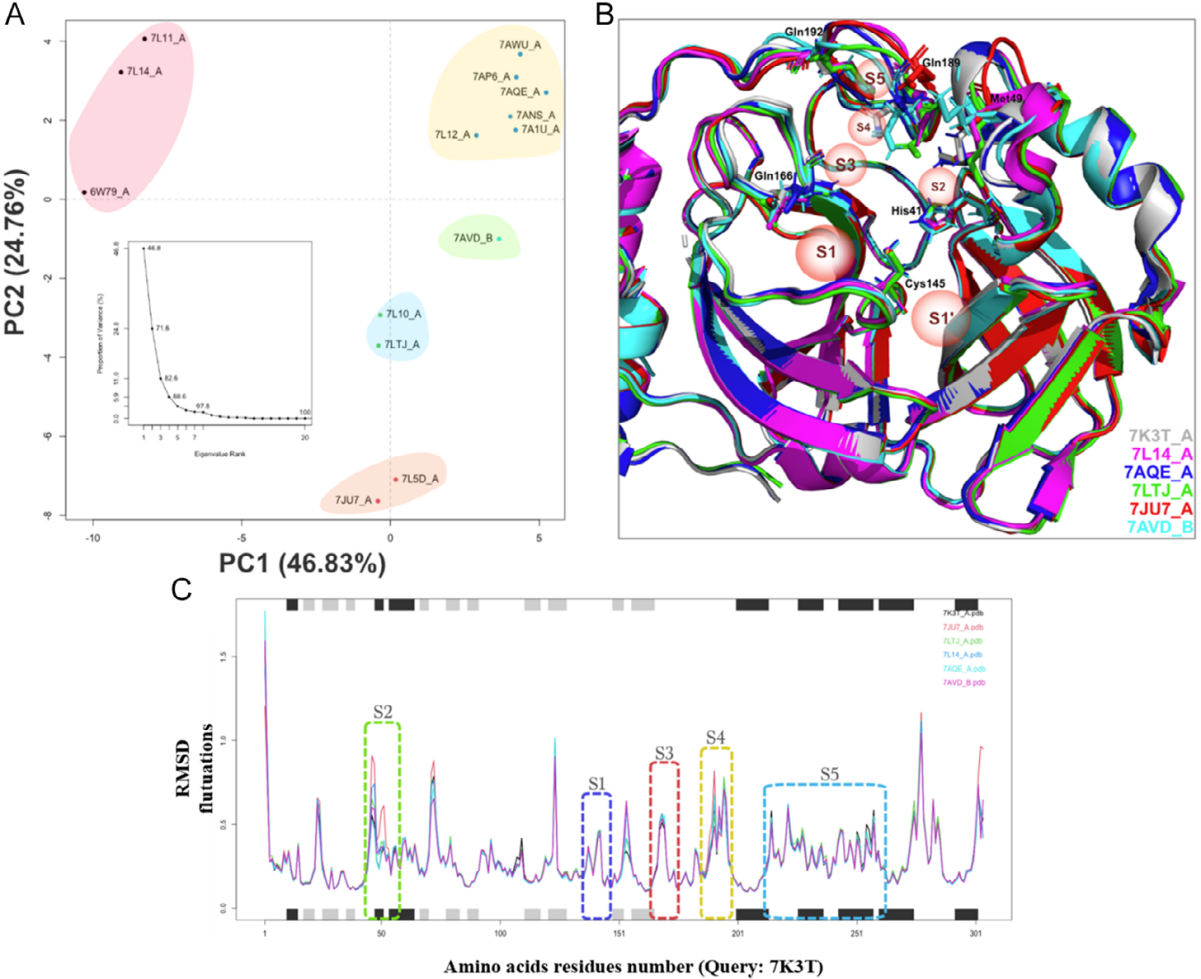
Principal component analysis (PCA) and normal mode analysis (NMA) for the selection of 3D structures of 3CL^pro^. Panel (A) presents the PCA analysis, with colors representing different clusters: red, yellow, green, blue, and orange. Panel (B) illustrates the conformational differences among amino acids, their respective subsites S1, S1’, S2, S3, S4, S5, and the organization of the canonical active site. Panel (C) displays the NMA, with dashed lines in green, blue, red, yellow, and cyan corresponding to subsites S1, S2, S3, S4, and S5, respectively. Continuous lines represent each 3D structure selected, with colors corresponding to PDB IDs 7K3T [33], IDs 7JU7 [34], 7LTJ [35], 7L14 [36], 7AQE [37], and 7AVD [37], respectively.

The conformational variability among these structures was assessed through principal component analysis (PCA) and NMA. PCA clustered the structures into five distinct groups, explaining 71% of the total variance, indicating the presence of unique structural conformations among them (Figure 1A). Based on this analysis, structures with PDB IDs 7JU7 [34], 7LTJ [35], 7L14 [36], 7AQE [37], and 7AVD [37] were selected and superposed (Figure 1B), reflecting their dynamic nature. Notably, subsites S2 and S4, which exhibited the highest RMSD fluctuations in NMA, also displayed conformational variations in S2, such as Cys44 (the highest variation of 1.8 Å) and Met49 (the highest variation of 4.9 Å), and in S4, such as Glu166 (the highest variation of 3.4 Å) and Gln189 (the highest variation of 3.4 Å). The structural variations were further characterized using NMA, which revealed regions exhibiting the greatest root mean square deviation (RMSD) fluctuations when comparing representative conformations from each cluster (Figure 1C), thereby highlighting areas of significant structural flexibility.

This integrative methodological approach underscores the diverse conformational states of 3CL^pro^, offering insights into the enzyme's structural plasticity. Importantly, the observed fluctuations in subsites S2 and S4 are not merely a consequence of crystallographic resolution but emerge from a systematic and criteria‐driven analysis combining large‐scale structural curation, PCA, and ensemble NMA. Such an approach enables the exploration of structure‐dependent protein flexibility and ligand‐induced adaptive motions, thereby providing a robust foundation for selection of representative receptor conformations.

### 2.3 Ensemble Docking Validation

The ensemble docking protocol was established and validated using the VS workflow in Maestro, Schrödinger [38]. All ensemble docking calculations were conducted within the canonical active site of 3CL^pro^, using a grid centered on the catalytic dyad (Cys145 and His41) (see *x*, *z*, *y* orientations in Table S3), for all PDBs selected from normal modes analysis (7AQE, 7AVD, 7JU7, 7L14, 7LTJ). The outcomes generated were subjected to statistical validation based on a panel of relevant scoring metrics, including ligand efficiency (LE) [39], Molecular Mechanics‐Generalized Born Surface Area (MM‐GBSA) free energy of binding [40], MM‐GBSA combined with LE, and Glide XP (Extra Precision) docking score [41]. Detailed information regarding these validation metrics can be found in Table S1.

MM‐GBSA calculations were performed as part of the validation analysis across the full set of docking poses, rather than being used as a final rescoring filter applied only to top‐ranked XP GlideScore poses. The statistical performance of MM‐GBSA and MM‐GBSA combined with LE was deemed suboptimal in effectively ranking known active compounds before inactive ones. Particularly, only the MM‐GBSA score yielded a moderately acceptable result, exhibiting an area under the receiver operating characteristic curve (ROC‐AUC) value of 0.70 across the entire ranked list. The integration of LE with MM‐GBSA did not demonstrably improve the enrichment of top‐ranked active candidates.

The statistical performance of the different scoring metrics was rigorously evaluated. While the LE metric also performed well (AUC of 0.85 and BEDROC of 0.80), the XP GlideScore was ultimately selected as the primary ranking metric. This decision was based on the XP GlideScore's slightly superior AUC (0.87) and BEDROC values, which indicated its enhanced ability to discriminate between active and inactive compounds, particularly at the top 10% of the list. Additionally, the XP GlideScore [41] incorporates a comprehensive set of physical energetic terms, including van der Waals and electrostatic interactions. This more detailed physical model provides a robust, reliable basis for ranking compounds and offers valuable insights into the binding mechanism, a significant advantage over metrics based on simpler calculations [41].

Conversely, LE is calculated by normalizing the GlideScore (without applying penalty terms) by the number of heavy atoms in the ligand molecule. Consequently, based on its superior performance in the validation analysis with the score, the XP GlideScore was selected as the primary metric for ranking the screened compounds and identifying the most promising candidates for further experimental evaluation.

### 2.4 Shape‐Based Models

**FIGURE 2:**
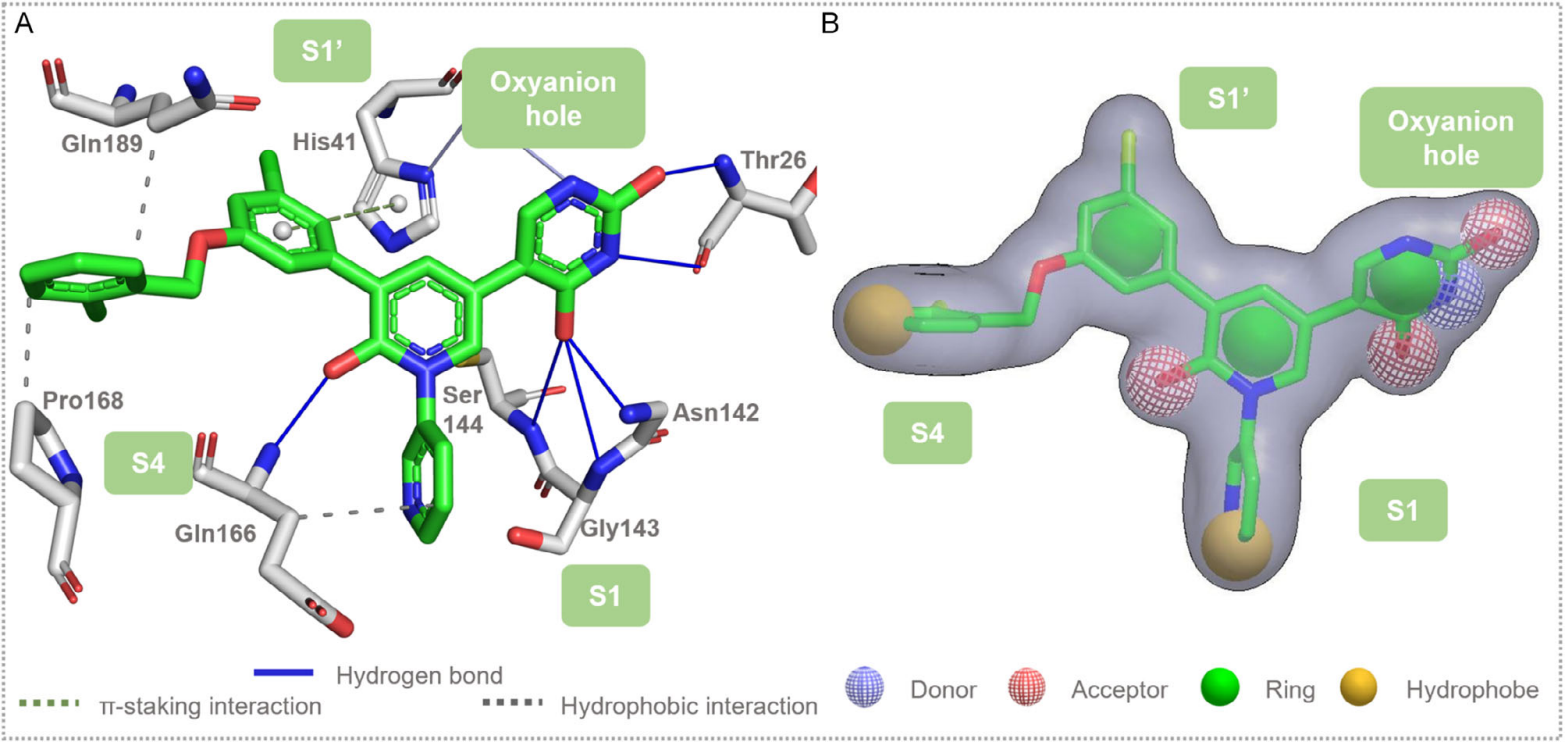
Structural basis for query generation. (A) Protein–ligand interaction profile of **compound 21** (PDB ID: 7L13 [36]) within the 3CL^pro^ catalytic site, generated via the PLIP platform [42]. (B) Resulting shape‐based model illustrating the molecular volume and pharmacophoric characteristics used for virtual screening (VS).

To evaluate the ability of shape‐based models to discriminate between active and inactive compounds, **compound 21** was used as a query (see 2D representation in Figure S2). Originally discovered by Zhang et al. [36], this noncovalent inhibitor exhibits an IC_50_ of 0.018 µM (FRET assay) and binds to the catalytic site as confirmed by X‐ray crystallography (PDB ID: 7L13; Figure 2A).

The compound establishes key binding interactions with the target protein, including π‐stacking with His41 in the S1′ subsite, hydrogen bonding with Thr26, Asn142, Gly143, Ser144 (located in the oxyanion hole and S1 subsite), and Gln166 (located in the S4 subsite), as well as hydrophobic contacts with Gln166, Pro168, and Gln189 (spanning the S1′ and S4 subsites).

Guided by these structural insights, we designed a shape‐based query to capture the essential pharmacophoric features (Figure 2B). The model incorporates: Hydrophobic regions derived from the chlorobenzene and pyridine rings; hydrogen‐bond acceptors from the carbonyl oxygens of the pyrimidine‐dione and pyridione moieties; a hydrogen‐bond donor from the pyrimidine‐dione nitrogen; and aromatic ring features that define the core molecular scaffold.

The performance of the generated models was assessed using RefTversky, RefTverskyCombo, and TanimotoCombo similarity scores. Statistical validation focused on enrichment at the top 1%, 5%, and 10% of the ranked library, which included actives, inactives, and decoys (Table 1). Among the evaluated functions, RefTversky demonstrated the most robust performance, achieving a ROC‐AUC of 0.87, an enrichment factor (EF) of 36.5, and a BEDROC value of 0.84 for the top 1% of the ranked list (Table 1). Given its superior ability to prioritize active compounds in the early enrichment phase, RefTversky was selected as the primary metric for the subsequent prospective screening.

**TABLE 1:** Statistical validation of shape‐based models.

Scores	AUC^a^	EF^b^_1%_	BEDROC^c^_1%_	EF_5%_	BEDROC_5%_	EF_10%_	BEDROC_10%_
**RefTversky**	**0.85**	**36.5**	**0.85**	**9.38**	**0.57**	**6.92**	**0.6**
RefTverskyCombo	0.79	38.5	0.84	8.85	0.55	6.35	0.57
TanimotoCombo	0.67	32.7	0.79	9.38	0.55	4.42	0.5

a AUC, Area under the curve.

b EF, Enrichment factors.

c BEDROC, Boltzmann‐Enhanced Discrimination of ROC at the top‐ranked 1%, 5%, and 10%, respectively.

While several similarity functions were evaluated, including TanimotoCombo, RefTverskyCombo, and RefTversky, a deeper analysis of the statistical metrics revealed that the RefTversky function was better suited for this VS campaign. Although TanimotoCombo yielded a respectable BEDROC of 0.79 in the top 1% of the ranked list, our chosen RefTversky model performed slightly better, with a BEDROC of 0.85. This marginal improvement, combined with the higher EF 1% of 36.5 for RefTversky compared to 32.7 for TanimotoCombo, indicated its superior ability to prioritize active compounds at the top of the list. The decision to prioritize the top 1% of the ranked list. However, a significant drop in BEDROC values was observed when changing the evaluation threshold to 5% or 10%. This trend signals that the ranking becomes less predictive and more susceptible to noise in the lower‐ranked portions of the database. This sensitivity highlights a common limitation in VS: while the models—including our ensemble docking (EF_1%_ = 7.03)—are highly effective for “early enrichment” and initial hit identification, their predictive power diminishes for compounds ranked outside the top percentile. To mitigate this impact, we adopted a high‐confidence selection strategy for our prospective screening, utilizing a strict 1% cutoff for the shape‐based stage to ensure only the highest‐quality candidates advanced to the subsequent ensemble docking and ML funnels.

Furthermore, a key advantage of the RefTversky function [43] is its flexibility in weighing the importance of different molecular features, including hydrogen bond donors and acceptors, hydrophobic regions, and aromatic rings. This contrasts with the Tanimoto function, which treats all elements uniformly. For our study, this tunability allowed us to emphasize pharmacophoric features critical for binding to the 3CL^pro^ active site, as identified in our structural analysis of the query compound, and to systematically explore different weighting schemes to optimize the model's predictive performance on our validation set.

### 2.5 Analysis of Activity Cliffs

A comprehensive AC analysis was performed as an essential prerequisite for developing the ML models. ACs are defined as pairs of structurally analogous compounds that exhibit substantial differences in biological potency [44, 45]. Such discontinuities can significantly challenge the capacity of ML algorithms to establish accurate structure–activity relationships and generalize across chemical space [46]. To systematically identify these features, we utilized the computational platform SARANEA [47].

**FIGURE 3:**
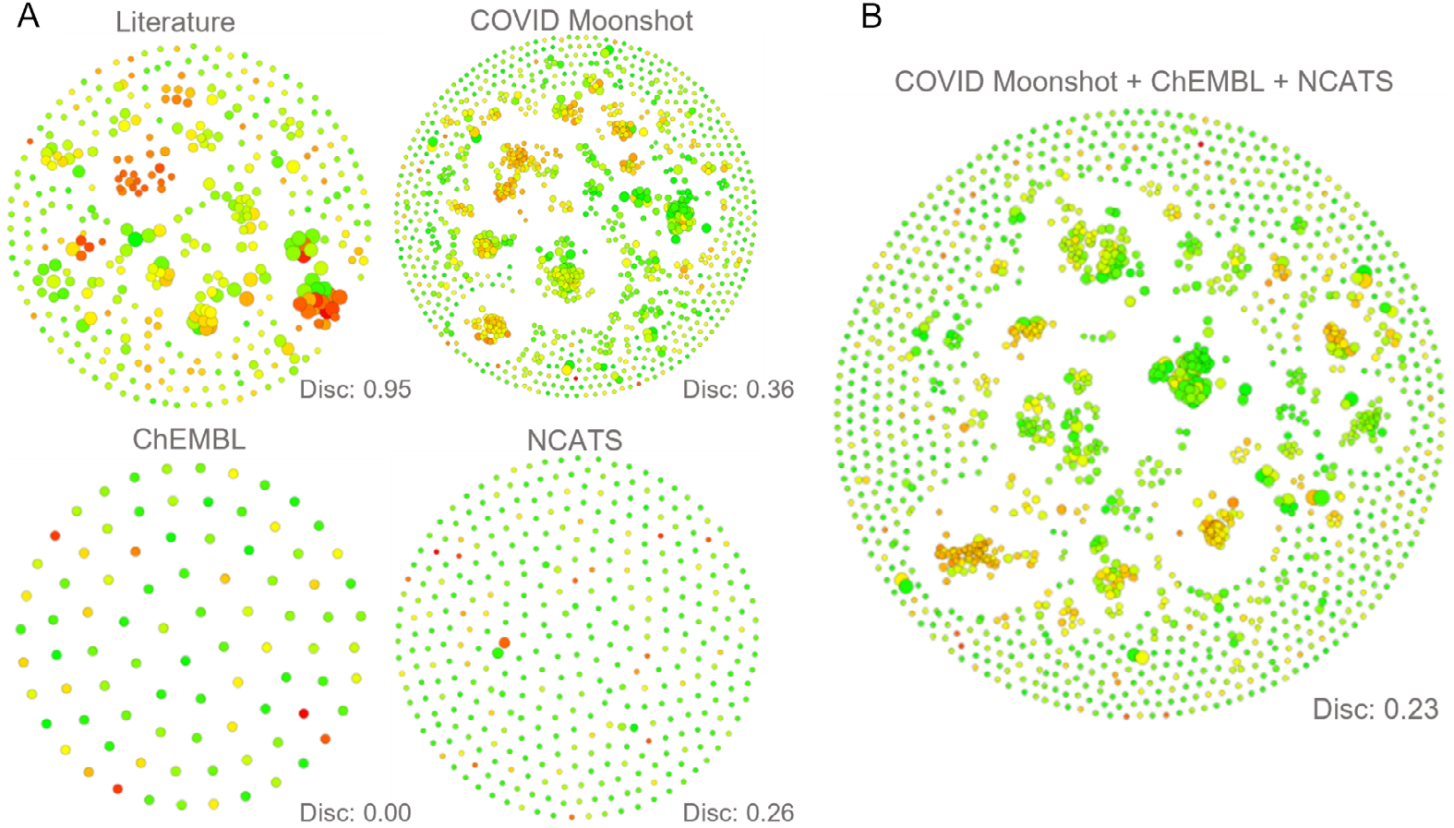
Structural similarity and activity cliff (AC) analysis generated via SARANEA [47]. (A) Comparative analysis of individual datasets (Literature, COVID Moonshot, ChEMBL, and NCATS). (B) Analysis of the integrated dataset following the exclusion of heterogeneous literature data. Spatial distribution: Nodes are organized using a force‐directed layout where physical proximity reflects structural similarity, resulting in the formation of scaffold‐based clusters. Node Color: Represents biological potency: green (IC_50_ > 10 μM); yellow (1 μM < IC_50_ ≤ 10 μM); red (IC_50_ ≤ 1 μM). Node size: Proportional to the individual discontinuity score; larger nodes highlight compounds involved in significant ACs, where minor structural modifications lead to substantial shifts in potency.

Figure 3 illustrates the individual and integrated dataset analysis. Within these networks, compounds are grouped into clusters based on shared structural scaffolds, with a color gradient representing potency (green: low; yellow: moderate; red: high potency). The discontinuity (Disc.) score provides a quantitative measure of these SAR discontinuities; higher values indicate that slight structural modifications result in disproportionate changes in activity [44, 47].

Disc. scores varied significantly across the datasets: 0.95 (Literature), 0.36 (COVID Moonshot), 0.0 (ChEMBL), and 0.26 (NCATS) (Figure 3A). The high score observed in the Literature dataset (0.95) highlights the critical impact of data heterogeneity. Although we prioritized bioactivity data derived from FRET assays to maintain experimental uniformity, the variability inherent in data originating from multiple laboratories remains a confounding factor.

As discussed by Ramírez‐Palma and Martinez‐Mayorga [48], such inconsistencies can introduce artifacts that mimic true SAR discontinuities. Furthermore, in the absence of experimental consistency, the fundamental assumption of a shared mechanism of action—a prerequisite for interpreting structural changes in the context of potency—cannot be reliably ensured. To ensure model reliability, the Literature dataset was excluded from further study. Merging the remaining high‐quality datasets resulted in a total discontinuity score of 0.23 (Figure 3B). This balanced score indicates a dataset with sufficient integrity for ML modeling, preserving the ability to learn from true SAR peaks while minimizing the noise introduced by experimental artifacts.

The identification of true ACs is highly sensitive to the quality and uniformity of the bioactivity data. To avoid the introduction of experimental artifacts—or “pseudocliffs”—we prioritized data from standardized FRET assays and excluded sources showing excessive discontinuity (e.g., the Literature dataset, Disc. = 0.95). By focusing on high‐quality, protocol‐consistent data, we ensured that the observed SAR peaks represent genuine molecular interactions rather than variations in assay conditions.

### 2.6 Machine Learning Models

**FIGURE 4:**
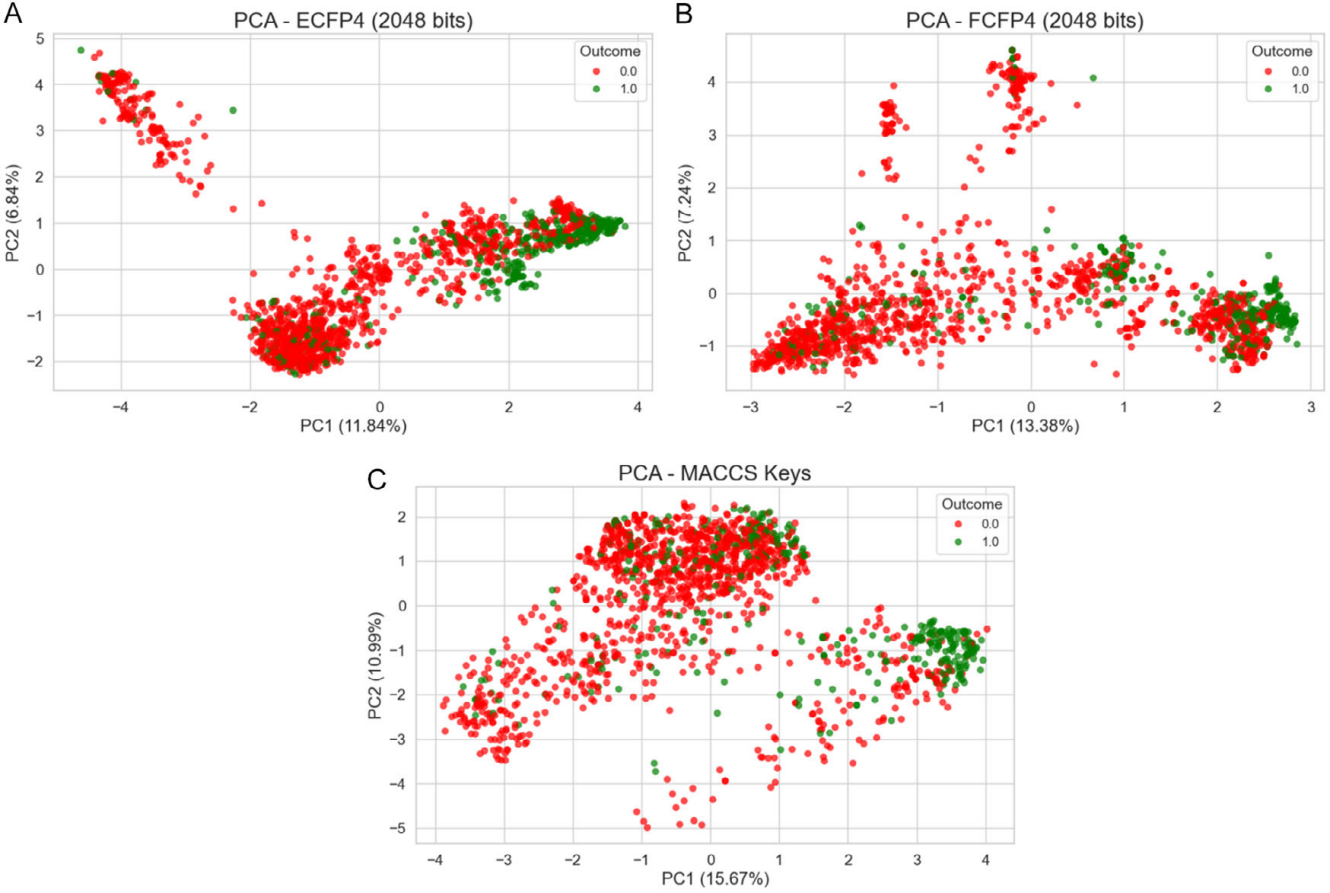
PCA analysis of ECFP4 (A), FCFP4 (B), and MACCS (C) molecular descriptors. The chemical space of inactive compounds is represented in red, and that of active compounds is depicted in green.

A chemical space analysis using PCA was conducted to support the selection of an appropriate molecular descriptor, comparing ECFP4 [49], FCFP4 [50], and MACCS keys [51] (166‐bits) (Figure 4). As expected, the high‐dimensional chemical space encoded by these fingerprints cannot be fully represented in a 2D projection. The variance explained by the first two principal components (PC1/PC2) was 11.84%/6.84% for ECFP4, 13.38%/7.24% for FCFP4, and 15.67%/10.99% for MACCS keys, respectively.

Despite the information loss inherent in this low‐dimensional approximation, the visual inspection of the PCA projections provided useful qualitative insights into descriptor performance. The ECFP4 analysis suggested a clearer separation between active and inactive 3CL^pro^ inhibitors compared to the other descriptors, indicating that its atom‐centered connectivity encoding captures structural features relevant to bioactivity. In contrast, FCFP4 showed a more intermingled distribution, implying that pharmacophore‐based features were less discriminative in this specific projection. Although MACCS keys accounted for a higher proportion of total variance, the significant overlap observed suggested that its fixed dictionary of generic substructures lacks sufficient resolution to distinguish the specific chemical modifications driving 3CL^pro^ inhibition. Collectively, these observations support the selection of ECFP4 as the most informative descriptor for this dataset.

Following this analysis, multiple predictive models were constructed using the ECFP4 [49] combined with three ML algorithms (Random Forest ‐ RF [52], Support Vector Machine ‐ SVM [53], and Light Gradient Boosting Machine ‐ LGBM [54]). Table 1 presents the optimal performance metrics achieved by each algorithm; the remaining algorithms are described in Supporting Information S4. These metrics were derived from a fivefold cross‐validation procedure and evaluated on a 20% test set, which was partitioned before cross‐validation. All developed models demonstrated comparable variance between the cross‐validation and test set, indicating the absence of significant model overfitting.

Based on a comparative evaluation of several ML models (see Table 2), the ECFP4_RF model was identified as the optimal predictive tool for discriminating between active and inactive inhibitors of the SARS‐CoV‐2 3CL^pro^. This model leverages the ECFP4 fingerprint to encode chemical structures and employs the RF algorithm for classification. The ECFP4_RF model demonstrated robust predictive performance on the test set, achieving a balanced accuracy of 0.83, a sensitivity of 0.70, a specificity of 0.96, an AUC of 0.83, and an MCC of 0.71. The selection of the RF algorithm was further substantiated by its inherent lower susceptibility to overfitting and the relative ease of its hyperparameter optimization compared to alternative algorithms such as LGBM. The optimized hyperparameters for the RF algorithm, which were determined through parameter optimizations, were as follows: Number of trees = 100, Maximum depth = 62, Minimum samples split = 2, Minimum sample leaf = 1, and Bootstrap = True. The integration of the ECFP4 descriptor, which effectively captures pertinent chemical features, with the RF algorithm underscores the suitability of the ECFP4_RF model for the prospective identification of novel 3CL^pro^ inhibitors.

**TABLE 2:** Statistical validation of ML models. The table presents balanced accuracy (Bal‐ACC), sensitivity (Se), specificity (Sp), area under the curve (AUC), and Matthew's correlation coefficient (MCC) for the different tested fingerprints and ML algorithms.

Model	Bal‐ACC^a^	Se^b^	Sp^c^	AUC^d^	MCC^e^
ECFP4_LGBM_cross‐validation	0.80	0.78	0.83	0.80	0.57
ECFP4_LGBM_test	0.82	0.80	0.84	0.82	0.60
**ECFP4_RF_cross‐validation**	**0.79**	**0.62**	**0.96**	**0.79**	**0.65**
**ECFP4_RF_test**	**0.83**	**0.70**	**0.96**	**0.83**	**0.71**
ECFP4_SVM_cross‐validation	0.78	0.61	0.94	0.79	0.62
ECFP4_SVM_test	0.79	0.65	0.93	0.76	0.62

a Bal‐ACC, Balanced accuracy.

b Se: Sensitivity.

c Sp: Specificity.

d AUC, Area under the curve.

e MCC, Matthew's correlation coefficient.

Based on these findings, the ECFP4_RF model was identified as the top‐performing predictive model due to its robust performance and resistance to overfitting. ECFP4's effectiveness in capturing relevant chemical space characteristics enabled the model to learn the underlying SARs. Although calibration methods were investigated, they did not enhance performance, likely due to the dataset's balanced nature, and were therefore deemed unnecessary.

Additionally, the PCA plots (Figure 4) visually represent the chemical space overlap between clas, the slight visual entanglement of classes is expected due to the linear nature of PCA dimensionality reduction on high‐dimensional binary fingerprints. The effective separation of active/inactive classes is strictly evidenced by the supervised RF model metrics (MCC of 0.62), which captures non‐linear patterns not fully visible in the first two linear principal components.

While we employed a standard fivefold cross‐validation protocol to assess model performance, we acknowledge recent guidelines [55] highlighting the importance of statistical replicability in ML benchmarks. Ash and collaborators recommend rigorous protocols, such as 5 × 5 repeated cross‐validation followed by hypothesis testing (e.g., repeated measures ANOVA), to better estimate performance variance and avoid optimistic biases inherent to single‐pass cross‐validation. Although our current workflow relied on standard metrics (ACC/AUC) rather than these extensive statistical sampling procedures, the “practical significance” of our models, defined as the ability to inform high‐stakes decisions [55], was ultimately confirmed by the prospective experimental validation. The identification of active compounds in the subsequent FRET assays demonstrates that the models successfully captured the relevant structure‐activity relationships, fulfilling the primary objective of the drug discovery campaign despite the use of classical validation metrics.

### 2.7 Model Interpretability

**FIGURE 5:**
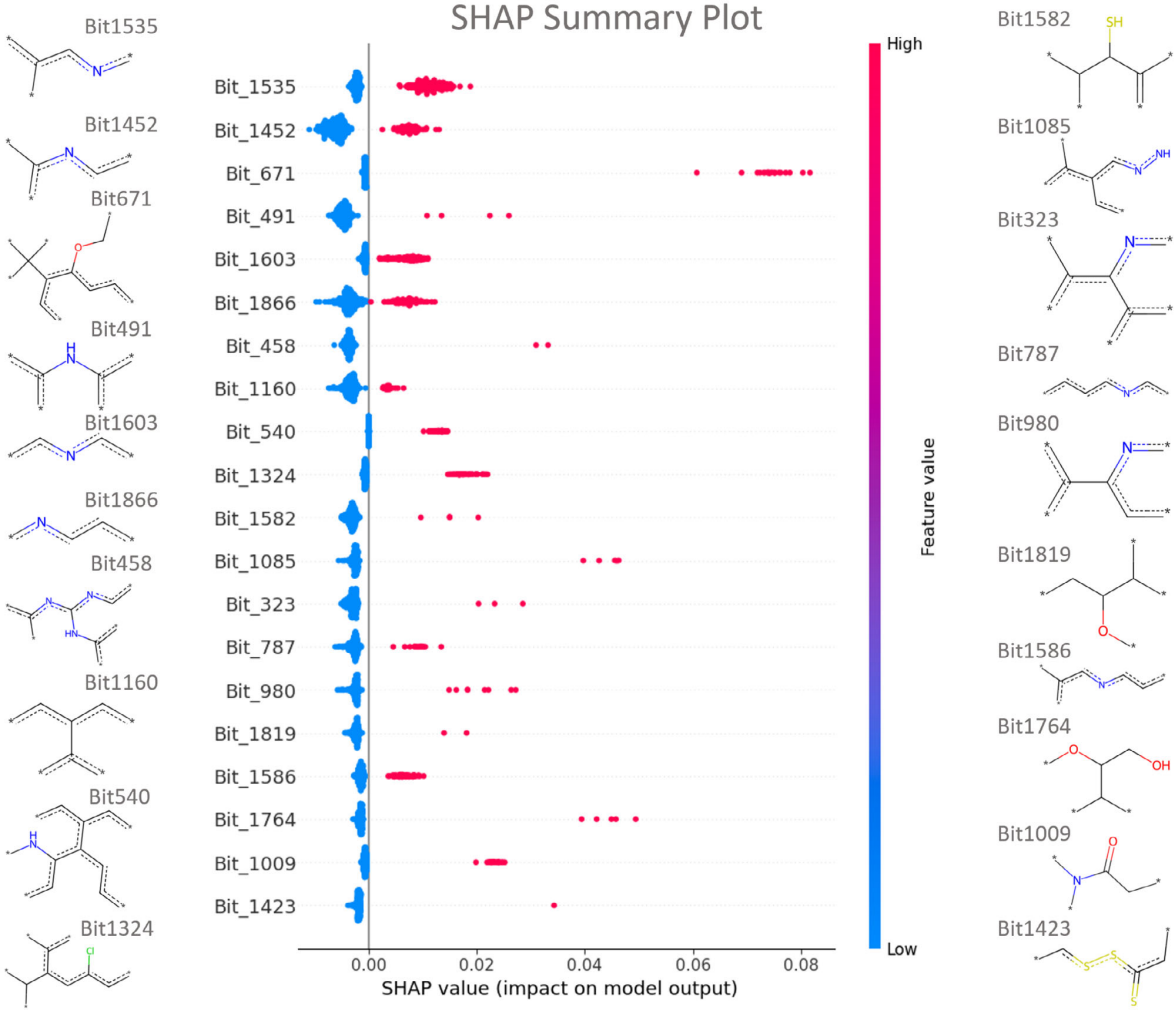
Feature importance analysis using SHAP values. SHAP summary plot showing the top 20 ECFP4 fingerprint bits ranked by their mean absolute SHAP value, indicating their contribution to the RF model's classification of active compounds. Each point represents the SHAP value of a given bit for an individual compound, with colors indicating low (blue) to high (red) feature values (bit absence or presence). Representative chemical substructures associated with selected bits (e.g., nitrogenous heterocycles, amides) are shown to aid interpretation of the pharmacophoric patterns learned by the model. High‐impact features such as Bits 1535 and 1452 are associated with pyridine‐ and pyrimidine‐like motifs, which typically mediate hydrogen bonding in the S1 pocket, while Bit 1324 reflects chlorinated aromatic motifs consistent with hydrophobic interactions in the S2 subsite.

To leverage the interpretability of the RF model and ensure that predictions were driven by chemically relevant motifs rather than artifactual correlations, we performed a SHAP (SHapley Additive exPlanations) analysis. This analysis identified the top structural features, represented by ECFP4 bits, that most strongly influence the classification of active 3CL^pro^ inhibitors (Figure 5).

As shown in Figure 5, the most significant features (e.g., Bits 1535, 1452, 1603, and 1866) are associated with nitrogen‐containing aromatic heterocycles, such as pyridine‐ and pyrimidine‐like substructures. These motifs are frequently observed in ligands that occupy the S1 pocket of 3CL^pro^, and are consistent with hydrogen‐bonding interactions involving residues such as His41, Gly143, and Ser144. Furthermore, the model identified linker regions as critical determinants of activity. Bits 491 and 1009 are associated with secondary amines and amide functionalities, respectively. These groups typically act as hydrogen bond donors and acceptors, mimicking the peptide bonds of the natural substrate and interacting with key backbone residues like Glu166.

Finally, hydrophobic and halogenated substructural patterns were also among the high‐impact features. Bit 1324 is associated with chlorinated aromatic motifs, whereas Bit 1160 reflects biaryl‐like system. These fragments are consistent with occupancy of the hydrophobic S2 subsite, where lipophilic and halogen‐mediated interactions contribute significantly to binding affinity. Collectively, the SHAP‐based interpretation indicates that the ML model captures chemically meaningful substructural patterns that are consistent with established structural and pharmacophoric requirements for SARS‐CoV‐2 3CL^pro^ inhibition.

### 2.8 Virtual Screening

**FIGURE 6:**
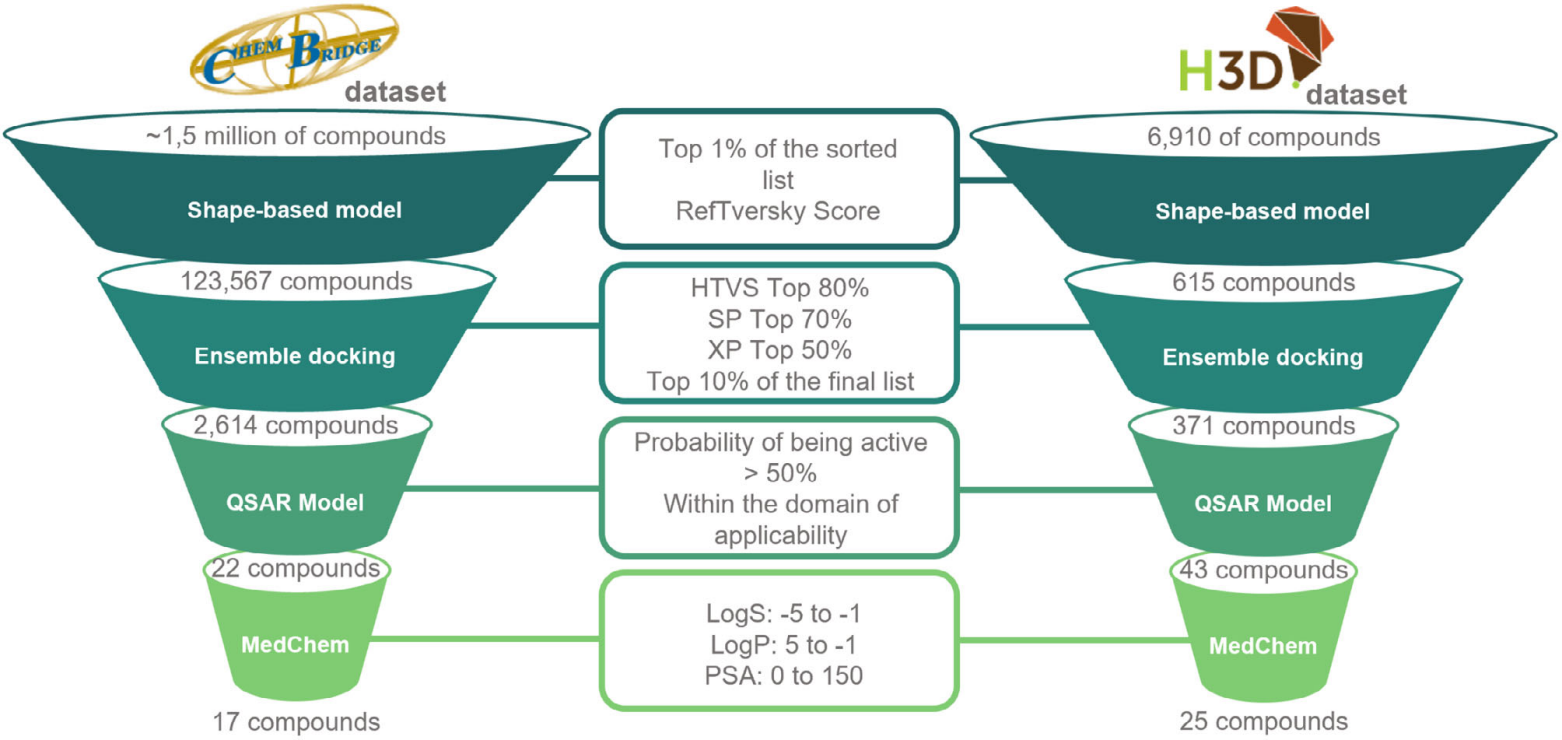
Hierarchical VS workflow for hit identification. The funnel illustrates the consensus strategy applied to the ChemBridge (left) and H3D (right) libraries. Stage 1 (Shape‐based): Prioritization of the top 1% of compounds based on 3D similarity to a known potent inhibitor (RefTversky score), specifically targeting pharmacophoric features relevant to 3CL^pro^. Stage 2 (Ensemble Docking): A multitiered mechanistic filter utilizing HTVS, SP, and XP modules to account for receptor flexibility across five representative 3CL^pro^ conformations. Stage 3 (QSAR/ML): Selection of compounds with a predicted activity probability >50% and within the applicability domain to ensure consistent physicochemical signatures. Final Selection: Medicinal chemistry evaluation based on established drug‐like properties (LogS, LogP, and PSA), resulting in the prioritization of candidates for experimental FRET and yeast‐based validation.

A VS campaign was conducted to identify potential 3CL^pro^ inhibitors using two different chemical databases (Figure 6): the ChemBridge database, which includes the EXP and CORE subsets totaling approximately 1.5 million compounds, and the University of Cape Town Holistic Drug Discovery and Development (H3D) Centre database, containing 6,910 compounds, a proprietary repository comprises a diverse collection of small molecules primarily synthesized during medicinal chemistry optimization campaigns for infectious diseases, including Malaria, Tuberculosis, and antimicrobial resistance (AMR). The library covers a broad chemical space, incorporating various chemotypes ranging from diversity‐oriented scaffolds to specific kinase inhibitors, all exhibiting drug‐like physicochemical properties suitable for biological screening. Both libraries underwent a three‐stage filtering process, utilizing the most accurate computational models developed in this study.

In the first stage, a shape‐based filtering model was applied. Compounds with the highest shape similarity to a known potent inhibitor (compound 21) were prioritized. Specifically, the top 1% of compounds from each database, ranked by the RefTversky score, were selected for further analysis. This resulted in 675 compounds from the H3D database and a much larger set of 123,567 compounds from the ChemBridge database.

Then, the selected compounds underwent ensemble docking using a VS workflow that incorporated the HTVS, SP, and XP [41] docking modules of Glide [41]. To minimize false negatives during early stages, the filtering was organized as the top 80% of compounds were retained after high‐throughput VS (HTVS), the top 70% after standard precision (SP) docking, and the top 50% after Extra Precision (XP) docking. From the final XP‐ranked list, a strict selection of the top 10% was performed for each database. This multitiered approach resulted in 371 prioritized compounds from the H3D database and 2264 from the ChemBridge database, which were subsequently subjected to MM‐GBSA rescoring and visual inspection.

In the third filtering stage, ML models were applied to prioritize compounds based on their predicted probability of activity (threshold > 50%) and their coverage within the models’ applicability domain. While the ML models were trained on a broader dataset that included covalent inhibitors to capture the fundamental molecular recognition signatures associated with 3CL^pro^ inhibition, this consensus strategy prioritized candidates that not only matched these potent physicochemical profiles but also fulfilled the strict geometric requirements of the noncovalent binding pocket identified via ensemble docking. By using ML as a broad structural filter and docking as a mechanistic gatekeeper, we ensured that the final candidates combined a global inhibitory “molecular signature” with the ability to form stable, reversible, noncovalent interactions. This step narrowed the selection to 43 compounds from the H3D database and 22 compounds from the ChemBridge database.

Following computational screening, all remaining compounds underwent a final medicinal chemistry evaluation focusing on their predicted physicochemical properties, including logS, logP, and PSA. The acceptable ranges, established from known active compounds, were as follows: LogS, −5 to −1; LogP, −1 to 5; and PSA, 0–1502 Å^2^, based on the known SARS‐CoV‐2 3CL^pro^ inhibitors compiled. Based on these criteria, 61 compounds were selected for experimental validation: 25 from the H3D database and 17 from the ChemBridge database.

This systematic, multifaceted computational approach, integrating shape‐based similarity, ensemble docking, ML predictions, and physicochemical profiling, ensures that only the most promising compounds are advanced to the experimental phase, thereby maximizing the likelihood of discovering effective 3CL^pro^ inhibitors with drug‐like properties.

### 2.9 Enzymatic Assays and Cellular Activity Tests in Yeast

The 42 selected compounds were subjected to biochemical enzymatic screening using FRET assays with 3CL^pro^. Based on the percentage of 3CL^pro^ activity, six compounds reduced the 3CL^pro^ biological activity to below 25% at 100 and 200 µM (Figure S3) and were subsequently evaluated for their half‐maximal inhibitory concentration (IC_50_). The corresponding dose‐response curves are presented in Figure S4, and Table 3 details the chemical structures and IC_50_ values of the six most promising compounds.

**TABLE 3:** Chemical structures and biological activity of the best compounds tested against SARS‐CoV‐2 3CLpro, using the FRET assay. The table presents the identification (ID), and half‐maximal inhibitory concentration (IC_50_) determined via FRET assays. Structural highlights indicate key motifs that align with the SHAP interpretability analysis. The established inhibitor N3 is included as a positive control (IC_50_ = 12.27 nM).

Structure	ID^a^	IC_50_, μM^b^	Chemical insight
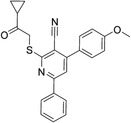	LabMol‐499	13.71 ± 1.34	• Contains nitrogenous heterocycles identified by SHAP (Bits 1535, 1452) for S1 subsite engagement. • Features 18 heavy aromatic atoms for π‐stacking/hydrophobic interactions. • Amenable to modification for solubility optimization without affecting allosteric binding.
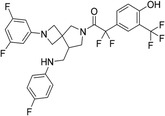	H3D‐009436	33.91 ± 4.27	• Incorporates secondary amine linkers identified as critical determinants (Bit 491). • Features multiple fluorine atoms and a ‐CF3 group (Bit 1324), mimicking hydrophobic patterns required for S2 subsite binding.
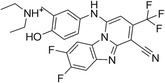	H3D‐008163	36.11 ± 1.34	• High nitrogen density (Bits 1535, 1452) consistent with S1 pocket H‐bonding with His163. • Contains a secondary amine linker (Bit 491) connecting the aromatic systems, facilitating substrate‐like orientation.
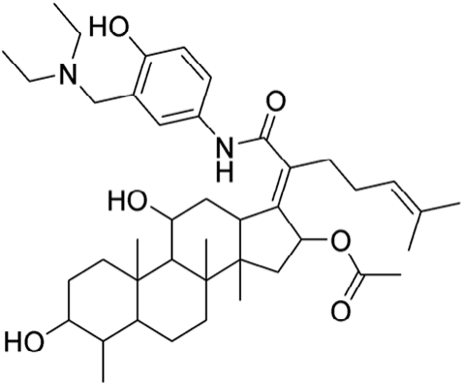	H3D‐005458	48.00 ± 19.35	• Represents a large biaryl‐like/lipophilic system (Bit 1160) targeting the hydrophobic S2 subsite. • Features an amide group (Bit 1009) that acts as a hydrogen‐bond acceptor/donor, mimicking peptide backbone residues.
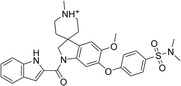	H3D‐009457	48.81 ± 6.80	• Features an indole‐based heterocycle (Bits 1535, 1452) for aromatic/polar interactions in the S1 subsite. • Includes a polar sulfonamide moiety and tertiary amine, providing H‐bond potential similar to peptide linkers (Bit 1009).
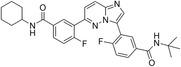	H3D‐000388	63.39 ± 15.59	• Contains a fused heterocyclic system (Bits 1535, 1452) targeting S1 residues. • Features two secondary amide functionalities (Bit 1009) that simulate peptide bonds for interaction with Glu166.
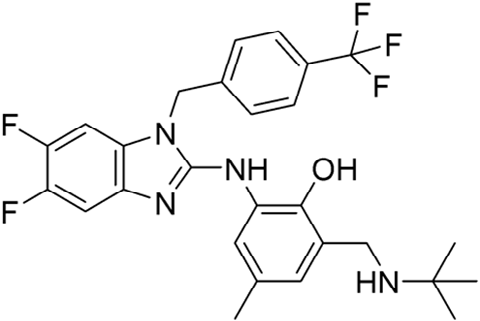	H3D‐007331	77.35 ± 31.48	• High impact from Bit 1324 via the ‐CF3 group and difluorinated ring, driving S2 subsite lipophilic interaction. • Provides nitrogen atoms (Bits 1535, 1452) for S1 pocket anchoring. • Employs a secondary amine (Bit 491) to maintain conformational flexibility and H‐bond donor capability.
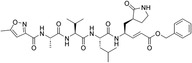	N3	0.01227	—

a Identification (ID).

b half‐maximal inhibitory concentration (IC_50_).

The inhibitory potencies of the selected compounds were quantitatively determined, revealing IC_50_ values ranging from 13.71 to 77.35 μM. Within the H3D series, H3D‐009436 (IC_50_ = 33.91 ± 4.27 μM) and H3D‐008163 (IC_50_ = 36.11 ± 1.34 μM) exhibited the most potent in vitro inhibitory profiles against SARS‐CoV‐2 3CL^pro^ compared to other structural analogs. Notably, the observed variability in IC_50_ values underscores the substantial impact of even subtle scaffold modifications on inhibitory potency.

As we can see from Table 3, LabMol‐499 emerged as the most potent hit, exhibiting an IC_50_ value of 13.71 ± 1.34 μM. The structural features of this compound closely mirror the high‐impact motifs identified by our SHAP interpretability analysis, particularly the nitrogen‐rich aromatic framework is strategically positioned to engage the S1 subsite. This alignment between ML‐based feature prioritization and experimental potency reinforces the validity of our integrated VS cascade.

**FIGURE 7:**
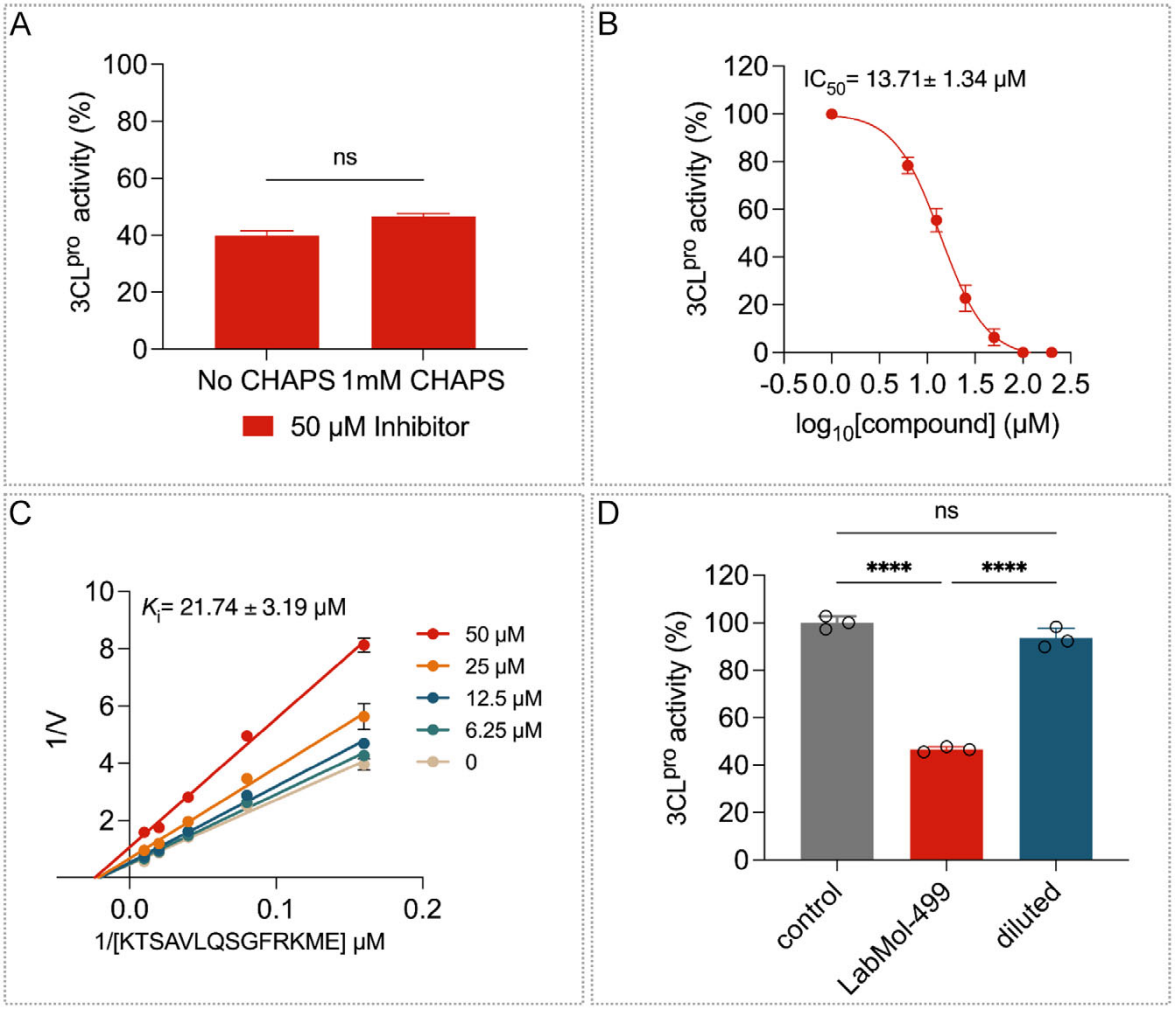
LabMol‐499 compound is a non‐competitive inhibitor of SARS‐CoV‐2 3CL^pro^ protease. (A) LabMol‐499 inhibition test in the absence and presence of 1 mM CHAPS detergent. (B) Dose‐response curve of LabMol‐499 in SARS‐CoV‐2 3CL^pro^ enzymatic activity. (C) Lineweaver–Burk plot of compound LabMol‐499 *K*_i_. (D) Assessment of compound activity reversibility by the jump‐dilution method.

To assess potential aggregation‐based inhibition, enzymatic assays were performed in the presence and absence of CHAPS (1 mM); no effect of CHAPS on 3CL^pro^ activity were observed, indicating that LabMol‐499 does not act as an aggregator (Figure 7A). Subsequently, a jump dilution assay was performed to determine the nature of the compound's binding to 3CL^pro^ [56]. This method is widely employed in early drug discovery to determine whether a compound inhibits its target via a reversible mechanism, a critical step in validating preliminary findings [57]. The inhibition constant (*K*_i_) of LabMol‐499 was determined to be 21.74 ± 6.7 μM (Figure 7C). The kinetic analysis showed a progressive decrease in *V*_max_ with increasing concentrations of LabMol‐499, while the apparent *K*_M_ remained essentially unchanged, consistent with a noncompetitive inhibition mechanism (Figure 6C).

Following preincubation of the enzyme with 50 μM LabMol‐499, a 60‐fold dilution was rapidly executed. The full recovery of enzymatic activity after following dilution demonstrates the reversible binding of LabMol‐499 to 3CL^pro^ (Figure 7D). The established inhibitor N3 was employed as a positive control in our FRET assays, exhibiting an IC_50_ of 12.27 nM. N3 is a peptidomimetic “suicide inhibitor” that covalently modifies the catalytic Cys145, acting through an irreversible, competitive mechanism [58]. In contrast, LabMol‐499 demonstrates a reversible, noncompetitive mode of action. This distinction is significant; while N3 targets the conserved active site directly by mimicking the transition state, LabMol‐499 exploits a distinct allosteric mechanism. This suggests that LabMol‐499 occupies a unique chemical space and binding pocket, potentially offering a complementary pharmacological profile useful for combination therapies or for targeting variants resistant to orthosteric inhibitors.

Although LabMol‐499 was prioritized via an ensemble docking protocol targeting the canonical active site (Cys145‐His41), our kinetic analysis reveals a noncompetitive mechanism, suggesting the compound likely engages an allosteric pocket rather than the orthosteric center. This discrepancy between in silico prediction and in vitro mechanism is a known phenomenon in SARS‐CoV‐2 3CL^pro^ discovery, where the enzyme's significant plasticity allows ligands to stabilize distal metastable states despite possessing features that fit the active site [37, 59, 60, 61]. For instance, compounds such as ebselen [62] and certain niclosamide derivatives [63] were initially modeled as active‐site binders but was later confirmed to exert inhibition via allosteric modulation at the dimerization interface (domains II–III) or surface pockets. The cyanopyridine‐2‐thione scaffold of LabMol‐499 contains a distinct electron‐rich, aromatic architecture that may favor these hydrophobic allosteric grooves, similar to other noncompetitive inhibitors that exploit the enzyme's dynamic ’breathing’ motions. Future X‐ray crystallography or molecular dynamics simulations will be required to map the precise allosteric binding event.

Moreover, selected compounds were screened for cellular activity against 3CL^pro^ using the proliferation of genetically engineered yeast as a readout [64]. As seen in Figure S5 and Table S2, LabMol‐499 displayed a signal at a level considered significant in a previous compound library screen [65]. This indicates that the compound is bioavailable and is active inside cells. It is important to note that the degree of intracellular activity of a molecule strongly depends on its uptake efficiency and biostability, which can vary widely between, e.g., yeast and mammalian cells. For pharmaceuticals, these parameters are normally optimized for mammalian cells, so it is commonly observed that EC_50_ values with yeast cells are higher.

### 2.10 ADME Prediction

**FIGURE 8:**
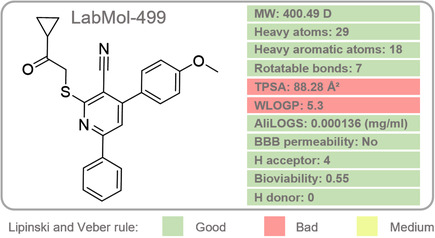
Predicted physicochemical and ADME profile of LabMol‐499. Schematic representation of key pharmacokinetic parameters obtained via the SwissADME platform. The compound exhibits favorable drug‐like characteristics, including a bioavailability score of 0.55 and adherence to Lipinski and Veber rules. The significant aromatic framework (18 heavy aromatic atoms) and high topological polar surface area (TPSA > 80 Å^2^) are consistent with its predicted lack of BBB permeability. Challenges regarding high lipophilicity (WLOGP = 5.3) and low aqueous solubility (AliLOGS) are identified as primary focal points for future hit‐to‐lead structural optimization.

The pharmacokinetic properties as absorption, distribution, metabolism, and excretion (ADME) of the best compound were predicted using the SwissADME platform [66] and are presented in Figure 8. The resulting physicochemical and drug‐likeness profiles provide critical insights into their potential as therapeutic candidates, highlighting key structural determinants of bioavailability and membrane permeability.

LabMol‐499 possesses a significant aromatic framework with 18 heavy aromatic atoms, which may facilitate target engagement through π‐stacking or hydrophobic interactions within the 3CL^pro^. The compound is predicted to exhibit limited permeability across the blood‐brain barrier (BBB), a characteristic consistent with its high topological polar surface area (TPSA > 80 Å^2^).

LabMol‐499's relatively high lipophilicity (WLOGP = 5.3) correlates with its low predicted aqueous solubility (AliLOGS = 0.000136 mg/mL), potentially posing a significant challenge to its progression. To address this, future optimization efforts will focus on strategic modifications to the scaffold, such as incorporating polar, hydrophilic groups to reduce the overall lipophilicity and improve aqueous solubility. Alternatively, exploring different salt forms or a hydrophilic prodrug strategy, where a polar moiety is temporarily attached to the molecule and later cleaved in vivo, can enhance its systemic availability without compromising the crucial binding interactions. The compound exhibits a predicted bioavailability of 0.55, indicating favorable absorption characteristics despite its solubility limitations. Crucially, as a noncompetitive inhibitor, the scaffold can tolerate greater structural modifications away from the canonical binding subsites (e.g., at the cyclopropyl‐ketone or the p‐methoxyphenyl moiety) to decrease WLOGP and increase aqueous solubility without necessarily compromising the key allosteric binding interactions.

## 3 Conclusion

This study successfully identified new hits active against the SARS‐CoV‐2 3CL^pro^, contributing to the ongoing quest for COVID‐19 therapies. The validated shape‐based model proved essential for guiding compound prioritization, while machine‐learning models combining ECFP4 descriptors with RF classifier provided reliable predictions of 3CL^pro^ inhibition, thereby streamlining early‐stage screening. VSs identified 43 promising compounds from commercial and collaborator‐sourced libraries, with FRET assays confirming seven with IC_50_ values below 80 μM. Notably, the identification of a noncompetitive mode of action for LabMol‐499 suggests that the compound likely binds to an allosteric site rather than the canonical active site. The noncompetitive kinetics provide a crucial rationale for future structural studies to pinpoint the allosteric binding pocket, thereby further expanding the druggable space of 3CL^pro^. Overall, this work emphasizes the importance of integrated and systematic screening and optimization in developing effective 3CL^pro^ inhibitors, with ongoing research promising significant contributions to global initiatives against SARS‐CoV‐2.

## Author Contributions

Each author has contributed significantly to this work. **Carolina Horta Andrade**, **I** **zabela Marques Dourado Bastos**, **Per Sunnerhagen,** and **Kelly Chibale** were responsible for securing the necessary funding. **Carolina Horta Andrade**, **Izabela Marques Dourado Bastos**, **Per** **Sunnerhagen**, **Richard Gessner**, and **Kelly Chibale** jointly conceived the study, provided overall direction, and supervision. **Sabrina Silva‐Mendonça**. performed the core computational work, including the development and validation of the shape‐based, ensemble docking, and machine learning models, and executed the final virtual screening and hit selection. **Fábio Muniz de Oliveira** carried out the selection and preparation of the 3CL^pro^ 3D structures (PDBs) for the ensemble docking analysis. **Carolina Horta Andrade** and **Melina Mottin** provided critical coordination and technical oversight for the computational procedures and analyses. **Donald Seanego** and **Christopher Jurisch** were responsible for the synthesis of the H3D compounds used for the experimental assays, with the coordination of **Richard Gessner**. **Flávia Nader Motta**, **Beatriz S. A.** **Rodrigues**, and **Gilberto S. M. Junior** performed and analyzed the 3CL^pro^ enzymatic and kinetic assays. **Sunniva Sigurdardóttir** and **Per Sunnerhagen** were responsible for the yeast cell‐based assay, where **Sunniva Sigurdardóttir** performed all experiments and analyzed the data. **Sabrina Silva‐Mendonça** was responsible for the initial data organization and wrote the first draft of the manuscript. All authors reviewed, edited, and approved the final version of the manuscript.

## Supporting Information

Additional supporting information can be found online in the Supporting Information section. **Supporting Information S1.** PDF file with Figure S1, S2, and S3, Table S1, S3, S3, and S4, as well as the detailed Materials and Methods and its references. **Supporting Information S2.** SDF file of the prepared compounds. **Supporting Information S3.** CSV file of the PDB structures compiled from PDB and the statistical metrics of each ML model generated. **Supporting** **Fig.** **S1**: General workflow for data collection, preparation, and standardization of chemical structures. **Supporting** **Fig. S2**: Bi‐dimensional representation of Compound 21. **Supporting** **Fig. S3**: Biological screening for Chembridge compounds in A, and H3D compounds in B. **Supporting** **Fig. S4**: The dose‐response curve for H3D selected compounds. **Supporting** **Fig. S5**: Growth curve of yeast cells expressing 3CLpro and a positive selection reporter plasmid^36^ with varying concentrations of A) compound 5924902 (300 μM: green, 100 μM; light blue, 10 μM: dark blue), or B) GC376 (50 μM: yellow, 10 μM: blue, 1 μM: purple). The curves represent the average of 4 replicates and standard deviation (each experiment was repeated 3 times). **Supporting** **Table S1**: Statistical validation of the ensemble docking model. **S** **upporting** **Table S2**: Statistical analysis of compound activity from growth curves shown below. **S** **upporting** **Table S3**: The X, Y, and Z orientations used on the grid generation for each selected PDB ID. **Supporting** **Table S4**: Statistical validation of classification machine learning models.

## Funding

This study was supported by CNPq (#440373/2022‐0, and #444750/2024−0), CNPq BRICS STI COVID‐19 (#441038/2020‐4), FAPEG (#202010267000272), CAPES (001), The Swedish Research Council (2020‐05738 and 2021‐03667), STINT (BR2018‐8017).  The South African Medical Research Council (SAMRC), Strategic Innovation Partnerships (SHIP) unit of the SAMRC, and the Technology Innovation Agency (TIA). The Wilhelm and Martina Lundgren Foundation. Carolina Horta Andrade and Izabela Marques Dourado Bastos are CNPq research fellows.

## Conflicts of Interest

The authors declare no conflicts of interest.

## Supporting information

Supplementary Material

## Data Availability

The data that support the findings of this study are openly available in GitHub at https://github.com/LabMolUFG/Structure‐‐and‐Ligand‐Based‐Discovery‐of‐Novel‐3CLpro‐Non‐peptidomimetic‐Hits and Zenodo at https://doi.org/10.5281/zenodo.17714716, reference number 17714716.
