# Supplementary material for: Structure‐ and Ligand‐Based Discovery of Novel 3‐Chymotrypsin‐Like Protease Nonpeptidomimetic Hits
Source: ChemMedChem. 2026 Apr 9;21(7):e202501083. doi: 10.1002/cmdc.202501083 (PMC13065885; doi:10.1002/cmdc.202501083)
Supplement: Supplementary file 1 — Supplementary Material [file CMDC-21-e202501083-s001.zip › Supp-Info-R1-chemmedchem-Mpro-.pdf]

SUPPLEMENTARY INFORMATION

Structure- and Ligand-Based Discovery of Novel $3CL^{pro}$ Non-peptidomimetic Hits

Sabrina Silva-Mendonça[a-c]; Donald Seanego[d]; Christopher Jurisch[d]; Melina Mottin[a-c]; Flávia Nader Motta[e-f]; Beatriz S. A. Rodrigues[e]; Gilberto S. M. Junior[e]; Alexandra Maria dos Santos Carvalho[e]; Fábio Muniz de Oliveira[a,g]; Sunniva Sigurdardóttir[g]; Per Sunnerhagen[h]; Izabela Marques Dourado Bastos[e]; Richard Gessner[d]; Kelly Chibale[d]; Carolina Horta Andrade[a-c, i]*.

[a] Dr. S. Silva-Mendonça, Dr. M. Mottin, Dr. F. Muniz de Oliveira, Prof. Dr. C. Horta Andrade*
Laboratory of Molecular Modeling and Drug Design (LabMol), Faculdade de Farmácia, Universidade Federal de Goiás, Goiânia, 74605-170, GO, Brazil
E-mail: carolina@ufg.br

[b] Dr. S. Silva-Mendonça, Dr. M. Mottin, Prof. Dr. C. Horta Andrade*
Center for the Research and Advancement in Fragments and Molecular Targets (CRAFT), School of Pharmaceutical Sciences at Ribeirão Preto, University of São Paulo, Ribeirão Preto, Brazil.

[c] Dr. S. Silva-Mendonça, Dr. M. Mottin, Prof. Dr. C. Horta Andrade*
Center for Excellence in One Health (CESU), Universidade Federal de Goiás, Goiânia, Brazil.

[d] Dr. D. Seanego, Dr. C. Jurisch, Dr. R. Gessner, Prof. Dr. K. Chibale.
Holistic Drug Discovery and Development Center (H3D), University of Cape Town, Rondebosch 7701, South Africa.

[e] Prof. Dr. F. Nader Motta, B. S. A. Rodrigues, G. S. M. Junior, A. M. dos Santos Carvalho, Prof. Dr. I. Marques Dourado Bastos.
Laboratory of Pathogen-Host Interaction (LIPH), Universidade de Brasília, Brasília, DF, Brazil.

[f] Prof. Dr. F. Nader Motta
Faculdade de Ceilândia, Universidade de Brasília, Brasília, Brazil.

[g] Dr. F. Muniz de Oliveira
McGowan Institute for Regenerative Medicine, Department of Surgery, University of Pittsburgh, Pennsylvania, 15219, USA.

[h] Dr. S. Sigurdardóttir, Prof. Dr. P. Sunnerhagen.
Department of Chemistry and Molecular Biology, University of Gothenburg, 40530 Gothenburg, Sweden.

[i] Prof. Dr. C. Horta Andrade*
Center for Excellence in Artificial Intelligence (CEIA), Institute of Informatics, Universidade Federal de Goiás, Goiânia, Brazil.

Table of Contents

Figure S1……………………………...………..……………………………………………..………..…...S3

Table S1..…………………………………………..……………………..……………......…...S4

Figure S2..………………………………………….....…………………………………...……....S5

Figure S3………………...………………………….………….......................……...…......S6

Figure S4……………...………………………..…………………………......………...…..……..S7

Table S2…………………………………………………………...…………......………...……....S8

Table S3……………………………………………………......…………………...…….………….S9

Materials and Methods……………………………………………………………………………S10

References……………………………………………………………………………………………S14

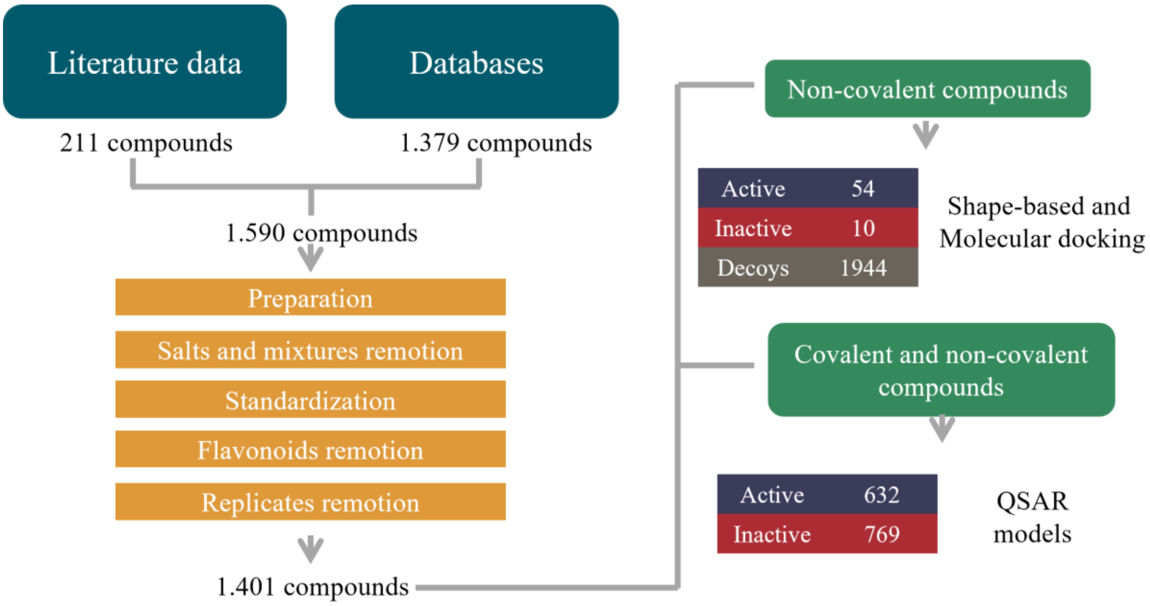

Figure S1. General workflow for data collection, preparation, and standardization of chemical structures.

Table S1. Statistical validation of the ensemble docking model.

Scores	AUC	$EF_{1\%}$	$BEDROC_{1\%}$	$EF_{5\%}$	$BEROC_{5\%}$	$EF_{10\%}$	$BEDROC_{10\%}$
MM-GBSA	0.70	9.38	0.31	3.12	0.2	2.84	0.25
MM-GBSA+LE	0.5	6.35	0.17	1.88	0.12	1.25	0.13
LE	0.85	34.38	0.80	9.69	0.56	5.78	0.62
**Glide XP**	**0.87**	**34.38**	**0.81**	**10.31**	**0.6**	**7.03**	**0.57**
MM-GBSA	0.70	9.38	0.31	3.12	0.2	2.84	0.25

AUC: Area under the curve. EF: Enrichment factors and BEDROC: Boltzmann-Enhanced Discrimination of ROC at the top-ranked 1%, 5%, and 10%, respectively.

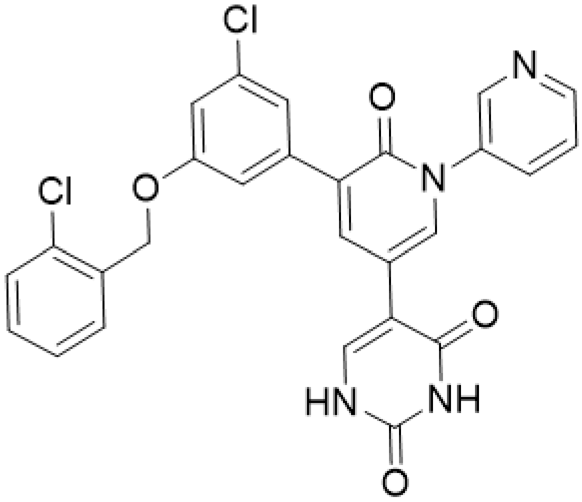

Figure S2. Bi-dimensional representation of Compound 21.

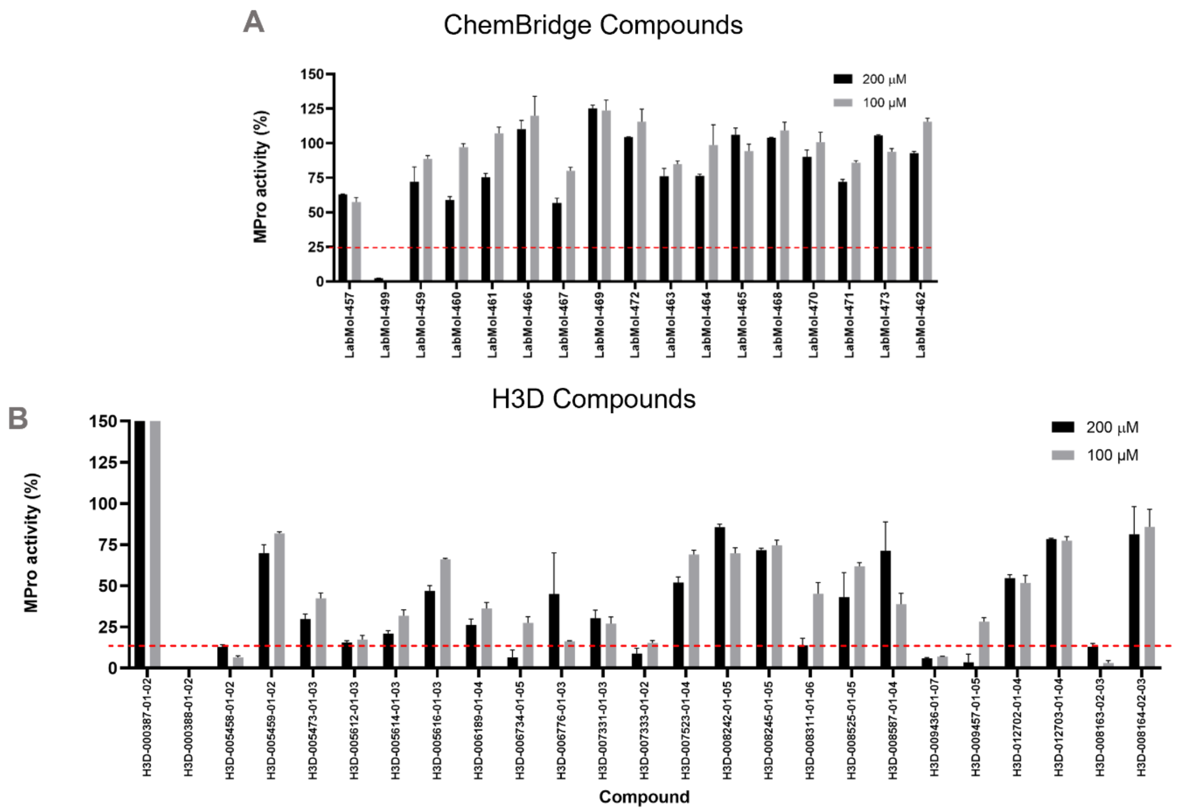

Figure S3. Biological screening for Chembridge compounds in A, and H3D compounds in B.

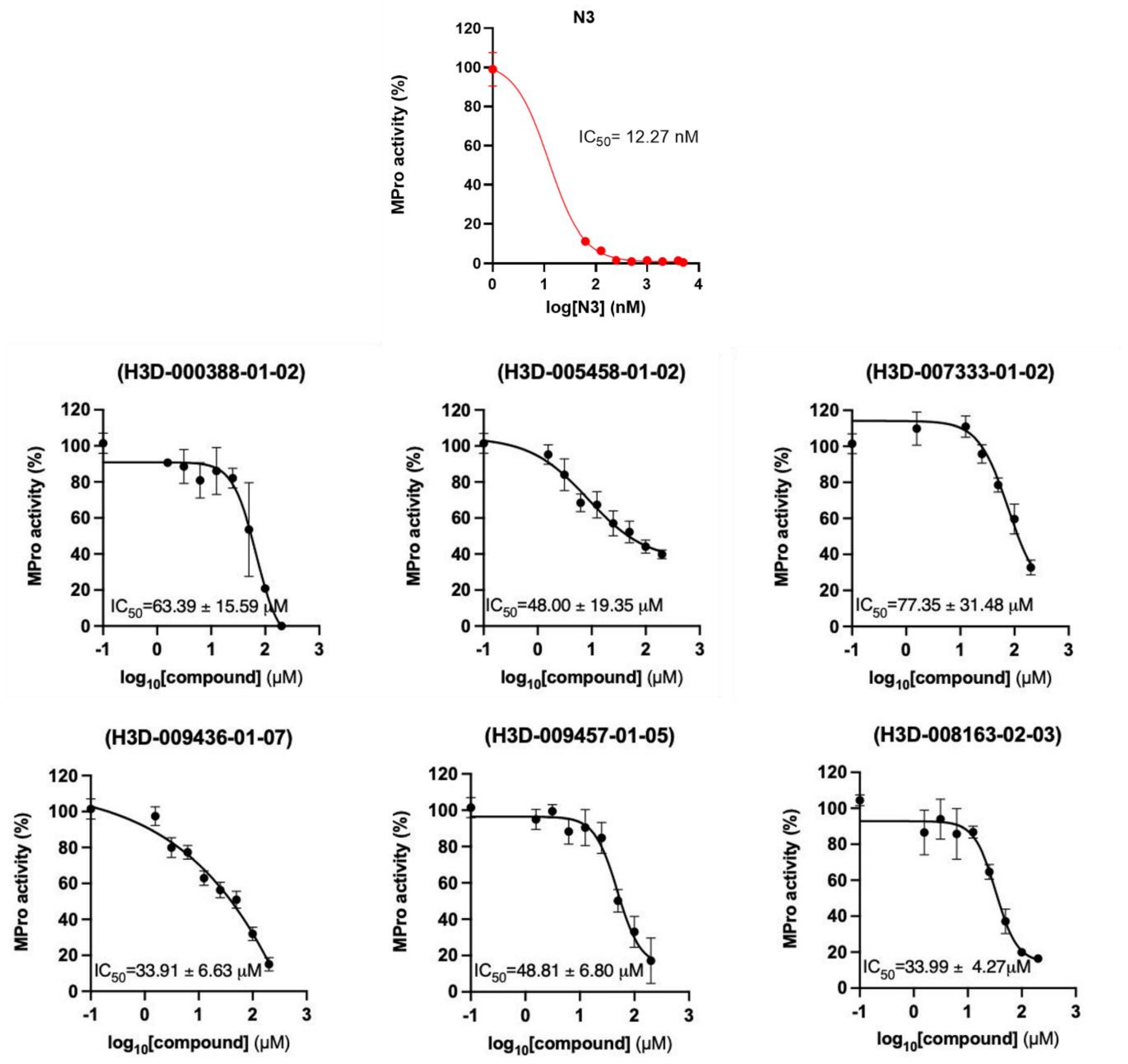

Figure S4. The dose-response curve for H3D selected compounds.

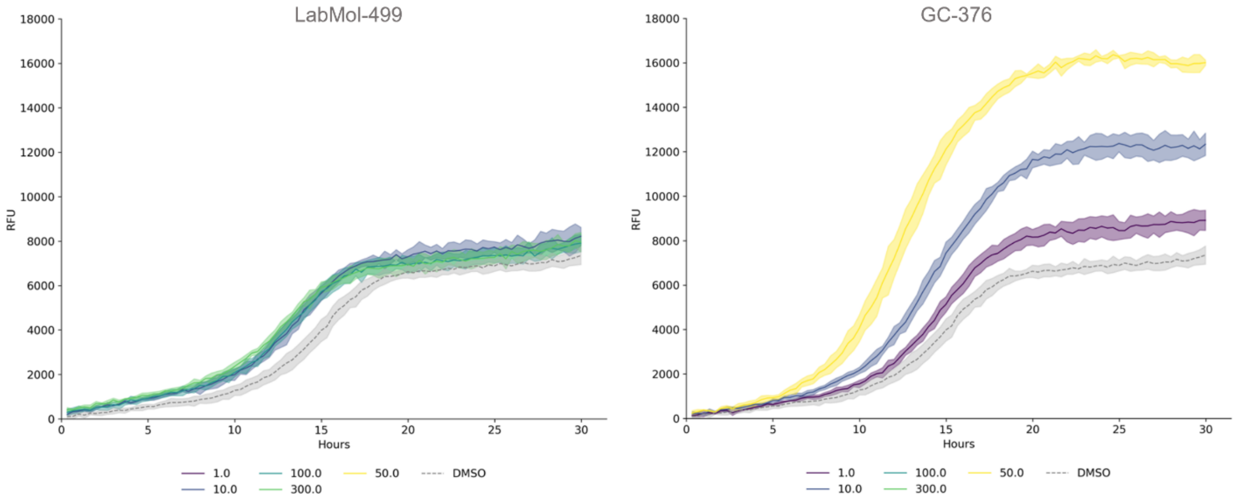

Figure S5. Growth curve of yeast cells expressing 3CLpro and a positive selection reporter plasmid[36] with varying concentrations of A) compound 5924902 (300 μM: green, 100 μM; light blue, 10 μM: dark blue), or B) GC376 (50 μM: yellow, 10 μM: blue, 1 μM: purple). The curves represent the average of 4 replicates and standard deviation (each experiment was repeated 3 times)

Table S2. Statistical analysis of compound activity from growth curves shown below.

COMPOUND	CONCENTRATION (µM)	AUC (FC) [1]	P-VALUE	ADJ. P-VALUE[2]
Labmol-499	10	1.13 ± 0.09	0.05	0.08
Labmol-499	100	1.10 ± 0.05	**0.02**	**0.03**
Labmol-499	300	1.13 ± 0.04	**0.004**	**0.008**
GC376	1	1.19 ± 0.08	**0.005**	**0.009**
GC376	10	1.48 ± 0.05	**> 0.001**	**> 0.001**
GC376	50	1.94 ± 0.03	**> 0.001**	**> 0.001**

1: AUC (FC): Fold change of the area under the curve compared to DMSO controls (average +/- SD, n = 4)

2: FDR adjustment (false discovery rate)

Table S3. The X, Y, and Z orientations used on the grid generation for each selected PDB ID.

PDB ID	X	Y	Z
7AQE	-18.1855514928800	3.50546126780000	3.41517973750000
7AVD	-1.95931320480000	-14.3909448733330	10.2694848687000
7JU7	-2.36180425090000	-11.5389301595400	8.35394192280000
7L14	-4.65066995990000	-14.3701659552920	7.08000677600000
7LTJ	-4.15000000000000	-13.4400000000000	5.92000000000000

Materials and methods

Protein structure selection

The best 3D structures from 3CL^{pro} were selected using Bio3D v.3.09[1], with RStudio v.1.1.46310 for Mac, R v.4.0.511 for Mac, and Bio3D-web[2]. Structures with gaps were removed, and clustering was performed using Root Mean Standard Deviation (RMSD) and Principal Components Analysis (PCA) methods in RStudio. The ensemble normal modes were then calculated using Bio3D-web. First, all experimental structures available in the Protein Data Bank[3] as of April 2021 were compiled. Second, all structures with poor resolution (<2 Å) were removed, and compounds that formed covalent bonds with 3CL^{pro} were excluded. After preparation, the resulting PDBs were submitted to Bio3D.

Data compilation, preparation, and standardization

A dataset of 3CL^{pro} inhibitors was manually curated from the literature and public databases. The compounds were prepared according to Fourches and collaborators[4,5]. The curation workflow involved removing inorganic counterions and mixtures, followed by structural cleaning (deionization), neutralization, and tautomer canonicalization to ensure chemotype consistency. Biological activity data were normalized to a standard unit (µM). A cutoff value of 10 µM was established to classify compounds as active (IC_{50} ≤ 10 µM) or inactive (IC_{50} > 10 µM), following the procedures of Unoh and collaborators[6]. Duplicate entries were analyzed to ensure label consistency: discordant pairs (same compound with both active and inactive labels) were removed from the dataset to prevent model confusion. For concordant duplicates (multiple entries with the same label), the entry with the most potent activity or the most recent report was retained, resulting in a unique entry per compound.

To validate the structure-based virtual screening models, a set of decoys was generated using the DUD-E methodology described by Shoichet and collaborators[7]. The workflow, implemented in KNIME 4.1[8], selected compounds from the ZINC13 *Drugs Now* database. Decoys were selected to match the physicochemical properties (molecular weight, logP, number of rotatable bonds, and hydrogen bond donors/acceptors) of the active non-covalent subset while ensuring topological dissimilarity (determined by a Tanimoto coefficient-based threshold). A ratio of 1:36 (actives:decoys) was applied to mimic the chemical space of a real high-throughput screening scenario, where inactives significantly outnumber active hits.

Shape-based models

The shape-based models were generated using ROCS (Rapid Overlay of Chemical Structures) module from OpenEye Scientific Software[9]. **Compound 21** (IC_{50} = 0.018 µM; PDB ID: 7L13)[10], was utilized as the reference query. To preserve the input protonation states, the ExplicitMillsDean force field was applied for all overlays. The validation library—consisting of the same actives, inactives, and decoys used in the ensemble docking—was screened to evaluate three distinct scoring functions: TanimotoCombo, RefTverskyCombo, and RefTversky.

The similarity between the query (*f*) and each database molecule (*g*) was quantified using the following metrics[11–13]. TanimotoCombo, a cumulative score ranging from 0 to 2, defined as the sum of

Shape Tanimoto (ST) and Color Tanimoto (CT). where ST measures global volume similarity and is calculated as the intersection over the union of volumes, as shown in equation 1:

$$\square\square = \frac{\square_{\square,\square}}{\square_{\square}} \quad (1)$$

Where $\square_{\square,\square}$ represents the overlap and $\square$ represents self-overlap. This metric penalizes size mismatches[11–13]**.** CT applies the same Tanimoto logic to quantify the overlap of pharmacophoric features (e.g., hydrogen-bond donors/acceptors, rings, and hydrophobes).

The RefTversky (Reference Tversky) is an asymmetric metric that quantifies volume overlap relative to the query's volume, as shown in equation 2:

$$\square_{\square\square\square} = \frac{\square_{\square,\square}}{\square_{\square} + \square_{\square} - \square_{\square,\square}} \quad (2)$$

Unlike the Tanimoto coefficient, RefTversky does penalize additional volume in the target molecule, provided the query's shape is well-embedded within the target scaffold[11–13].

The RefTverskyCombo is analogous to TanimotoCombo: it sums the Shape RefTversky (SR) and Color RefTversky (CR) components (SR + CR), yielding a value between 0 and 2.

Validation performance was assessed using the same statistical metrics employed for docking (ROC-AUC, EF, and BEDROC) to ensure consistency across the virtual screening workflow.

Protein, compounds, and grid preparation

The Protein Preparation Wizard program[14,15] prepared the 3CL^{pro} structures, and the ligands were prepared using the LigPrep program[14,16] within the Maestro v.12.9 platform[14,16] from Schrödinger[17]. The hydrogen atoms were added, bond order and formal charges were adjusted, and the amino acid protonation state was predicted at a neutral pH of 7.3 ± 0.5 using Epik v. 4.3[18,19] Grid generation in Maestro v. 11.5, from Schrödinger, was used to set the grid in the five prepared protein structures. All proteins were aligned before calculation, and the X, Y, and Z orientations are presented in Supporting Information Table S2.

Ensemble docking

Ensemble docking was performed using the Virtual Screening Workflow (VSW) in Maestro v. 11.5 (Schrödinger, LLC) with the Glide program[20–23]. To account for receptor conformational flexibility, five representative crystal structures of SARS-CoV-2 3CLpro were utilized (PDB IDs: 7AQE[24], 7AVD[24], 7JU7[25], 7L14[26], and 7LTJ[27]). The protein structures were prepared using the Protein Preparation Wizard, including bond order assignment, hydrogen addition at pH 7.4, and energy minimization using the OPLS3 force field. Docking grids were generated for each protein conformation by centering the grid box on the catalytic dyad residues Cys145 and His41, ensuring consistent coverage of the canonical active site across the ensemble.

The docking protocol was validated retrospectively using a curated dataset comprising 54 non-covalent active inhibitors and 1,944 property-matched decoys (generated via DUD-E methodology, ratio 1:36), as detailed in Supplementary Information Table S3. All ligands were docked

against all five protein conformations following a hierarchical docking workflow consisting of High-Throughput Virtual Screening (HTVS), Standard Precision (SP), and Extra Precision (XP). Ensemble aggregation was performed using a best-score strategy, in which each ligand was assigned the most favorable score obtained across the five protein conformations.

To optimize hit identification while assessing model robustness, two distinct filtering protocols were employed. For the validation study (benchmarking), a conservative approach was adopted to enable a comprehensive analysis of enrichment metrics: the entire dataset (actives and decoys) was sequentially docked using HTVS, SP, and XP modes, retaining 100% of successfully docked compounds at each stage. This enabled the calculation of complete ROC curves and unbiased metrics, Enrichment Factor (EF 1%, 5%, and 10%)[28,29], Area Under the ROC Curve (AUC)[30], and BEDROC[31], without cutoff bias.

In contrast, for the prospective virtual screening of the H3D and ChemBridge libraries, a stepwise filtering strategy was implemented to maximize computational efficiency. Compounds were progressively filtered based on their ensemble rankings: the top 80% were retained after HTVS, the top 70% after SP, and the top 50% after XP. From the final XP-ranked list, a strict selection of the top 10% best-scoring compounds was applied. Finally, the binding free energy was estimated using MM-GBSA[32] and Ligand Efficiency (LE) for the final XP poses to guide the visual inspection and hit prioritization.

Machine Learning models

Molecular fingerprints were calculated to represent the absence or presence of specific structures in the chemical structure. The fingerprints tested were ECFP4[33] and FCFP4[34,35] with 2048 bits and MACCS keys[36] (166-bits), using the open-source package RDKit[35] in Python v. 3.7.46[37].

The dataset was split into two parts: a test set, which accounted for 20% of the entire dataset, and a modeling set, which comprised 80%. The purpose of the test set was to assess the model's predictive performance, while the modeling set was utilized in a 5-fold cross-validation approach[38]. In this strategy, the modeling set is divided into five subsets of equal size that do not overlap. Four subsets were designated for the training set, and one was the validation set. This process was repeated five times, allowing for subsequent rotations of subsets as the validation set[39].

The ML models were calculated using machine learning methodologies in combination with fingerprints. The algorithms used were Support Vector Machine (SVM)[40], Random Forest (RF)[41], and Light Gradient Boosting Machine (LGBM)[42], which are available in the Scikit-learn v.0.19.2.47 library[43].

The parameters were optimized using the Bayesian optimization package in Scikit-Optimize v. 0.8.1 library[44]. Bayesian optimization identifies the primary function with the best hyperparameter values and replaces it with a new probabilistic function based on previous evaluations of hyperparameters[45]. The primary function chosen was the G-mean score due to a better balance between the majority (inactive) and minority (active) classes[45]. The parameters submitted to optimization for RF were: max_features: ['auto', 'sqrt'], threes: [100, 1000], max_depth: [2, 100], min_samples_leaf: [1, 20], min_samples_split: [2, 20]; for SVM: C: [1e-6, 1e+6, 'log-uniform'], gamma:

[1e-6, 1e+1, 'log-uniform'], kernel: ['rbf']; and for LGBM: learning_rate: [0.01, 1.0, 'log-uniform'], num_leaves: [7, 4095], n_estimators: [100, 800], max_depth: [2, 63], subsample: [0.4, 1], scale_pos_weight: [1, 1000].

The QSAR models were validated using the Sensitivity (Se), Specificity (Sp), Balanced Accuracy (BACC), and Matthews Correlation Coefficient (MCC), following the equations, respectively:

$$Se = \frac{TP}{(TP+FN)} \quad (3)$$

$$Sp = \frac{TN}{(TN+FP)} \quad (4)$$

$$BACC = \frac{(TP+TN)}{FN} \quad (5)$$

$$MCC = \frac{(TP*TN-FP*FN)}{\sqrt{(TP+FP)(TP+FN)(TN+FP)(TN+FN)}} \quad (6)$$

TP: True Positive; FN: False Negative. TN: True Negative; FP: False Positive; FN: False Negative.

To evaluate the reliability of each ML model, the applicability domain was calculated using the Dice similarity coefficient (Ds), following the same strategies described by Gadaleta and collaborators[46].

Preparation of Screening Libraries

Prior to virtual screening, the external datasets (H3D library and ChEMBL subset) were processed according to the specific requirements of each modeling strategy. For the Machine Learning (LBVS) approach, the datasets underwent the same curation protocol applied to the training set to maintain consistency with the specific chemotypes learned by the models. Chemical structures were standardized in 2D format, removing salts and mixtures, deionizing, and tautomer canonicalization, as described by Fourches and collaborators[4].

Conversely, in Docking and Shape-Based Screening (SBVS), an accurate 3D representation was prioritized. The 2D structures were converted to 3D, and protonation states were generated at physiological pH (7.4 ± 0.5) using LigPrep (Schrödinger)[16] and QUACPAC (OpenEye)[47], with tautomeric states optimized to the most probable forms in solution. Crucially, regarding stereochemistry, compounds with undefined chiral centers were enumerated to generate all possible stereoisomers (up to 32 per ligand), ensuring that potential bioactive isomers were not excluded from the screening. Finally, low-energy conformers were generated using OMEGA (OpenEye)[48,49] to facilitate the shape-based alignment.

Virtual screening and compound selection

The virtual screening was performed using the H3D library, a private database of compounds (comprising 6,910 compounds) from the University of Cape Town and the ChemBridge commercial database. The first filter is the best shape-based model and was selected in the top 10% of

compounds by RefTversky score, aiming to find new hits of 3CL^{pro}. Then, the second filter was the ensemble docking screening, which selected the top 10% of compounds from the virtual screening workflow, based on XP-GlideScore and MMGBSA scores, across HTVS, SP, and XP steps. The third, the best QSAR model evaluated, chose only compounds with a probability greater than 0.5 and inside the probability domain. The compounds selected from the H3D database were shared with the University of Cape Town, and the ChemBridge database was bought for distribution to enzymatic and kinetic assays.

Chemicals

The ChemBridge compounds were obtained from ChemBridge (San Diego, CA, USA) in a solid, salt-free form with a purity of ≥ 95%. Additionally, compounds from the Holistic Drug Discovery and Development (H3D) database, designated as H3D-0000388, H3D-0007331, H3D-0009436, H3D-0005458, H3D-0009457, and H3D-0008163, were synthesized as previously described: H3D-0000388, H3D-0005458, and H3D-0009457 by Le Manach and collaborators[50], H3D-0007331 by Leshabane and collaborators[51], Le Manach and collaborators[52], and H3D-0008163 by Dziwornu and collaborators[53].

3CL^{pro} expression, purification, enzymatic, and kinetic assays; growth-based activity assay in yeast

The recombinant SARS-CoV-2 3CL^{pro} was produced as previously described with some modifications. SARS-CoV-2 3CL^{pro} was cloned into pGEX-6P-1 and was transformed into Escherichia coli strain BL21(DE3). The expression of the 3CL^{pro} recombinant protein was induced by 1 mM IPTG for 18 hours at 18 °C. To purify the recombinant enzyme, cells were harvested, lysed with BugBuster® (Merck), and centrifuged at 16,000 g for 20 min at 4 °C. The supernatant was subjected to affinity chromatography using a nickel-agarose resin (HIS-Select HF Nickel Affinity Gel, Sigma). After the resin had been extensively washed with 50 mM Tris-HCl (pH 8.0), 0.5 M NaCl, and 5 mM imidazole, the bound recombinant enzyme was eluted with the same buffer containing 80 mM imidazole. The purified enzyme was dialyzed against 20 mM Tris-HCl, pH 7.3, concentrated, and stored at 4 °C.

Inhibition assays were performed in triplicate using 57 nM SARS-CoV-2 3CL^{pro} in 20 mM Tris-HCl (pH 7.3) and 1 mM EDTA for 15 min. The substrate Dabcyl-Lys-Thr-Ser-Ala-Val-Leu-Gln-Ser-Gly-Phe-Arg-Lys-Met-Glu (Edans)-NH_2 was added to a 100 µL reaction mixture at a final concentration of 20 µM. Fluorescence was subsequently measured at an excitation wavelength of 340 nm and an emission wavelength of 490 nm in kinetic mode for 20 minutes using the SpectraMax M5 Microplate Reader (Molecular Devices). IC_{50} values were calculated by fitting the curves to a four-parameter logistic (4-PL) non-linear regression model using GraphPad Prism® 8 software. Ki values were determined from five different inhibitor and substrate concentrations (100 – 6.25 µM) in 20 mM Tris (pH 7.3), 1 mM EDTA, and 1 mM CHAPS. All kinetic parameters were analyzed using the enzyme kinetics module of GraphPad Prism® and Hanes-Woolf plots.

Finally, a jump dilution analysis was performed to evaluate the reversibility of inhibition. Briefly, enzyme-inhibitor complexes were formed by preincubating $3CL^{pro}$ with inhibitors at 4× IC_{50} for 15 min at room temperature in a 12 µL reaction volume. After preincubation, the complexes were diluted 60× in 20 mM Tris (pH 7.3), 1 mM EDTA, with or without 1 mM CHAP. The substrate Dabcyl-Lys-Thr-Ser-Ala-Val-Leu-Gln-Ser-Gly-Phe-Arg-Lys-Met-Glu (Edans)-NH_2 was then added to a 100 µL reaction mixture at a final concentration of 20 µM. Activity detection and analysis were performed as described above. Data was analyzed using one-way ANOVA in GraphPad Prism® version 8 software.

Growth-based activity assays in yeast cells used a previously described principle[54]. Briefly, yeast was engineered to express $3CL^{pro}$ and a fusion protein consisting of the *E. coli* toxin MazF joint through a synthetic linker peptide to its cognate inhibitor MazE. A cleavage site for $3CL^{pro}$ was inserted into the linker. Inhibition of $3CL^{pro}$ by an exogenous small molecule result in reduced protease activity, leading to less liberated free MazF and increased cell proliferation. Yeast cell proliferation was recorded from growth curves measured in a BMG Polarstar Omega fluorescence plate reader and quantified as described[55].

References

[1] B. J. Grant, L. Skjærven, X.-Q. Yao, “The Bio3D packages for structural bioinformatics” *Protein Sci.* **2021**, *30*, 20–30.

[2] L. Skjærven, S. Jariwala, X.-Q. Yao, B. J. Grant, “Online interactive analysis of protein structure ensembles with Bio3D-web” *Bioinformatics* **2016**, *32*, 3510–3512.

[3] H. M. Berman, J. Westbrook, Z. Feng, G. Gilliland, T. N. Bhat, H. Weissig, I. N. Shindyalov, P. E. Bourne, “The Protein Data Bank.” *Nucleic Acids Res.* **2000**, *28*, 235–242.

[4] D. Fourches, E. Muratov, A. Tropsha, “Trust, But Verify: On the Importance of Chemical Structure Curation in Cheminformatics and QSAR Modeling Research” *J. Chem. Inf. Model.* **2010**, *50*, 1189–1204.

[5] D. Fourches, E. Muratov, A. Tropsha, “Trust, but Verify II: A Practical Guide to Chemogenomics Data Curation” *J. Chem. Inf. Model.* **2016**, *56*, 1243–1252.

[6] Y. Unoh, S. Uehara, K. Nakahara, H. Nobori, Y. Yamatsu, S. Yamamoto, Y. Maruyama, Y. Taoda, K. Kasamatsu, T. Suto, K. Kouki, A. Nakahashi, S. Kawashima, T. Sanaki, S. Toba, K. Uemura, T. Mizutare, S. Ando, M. Sasaki, Y. Orba, H. Sawa, A. Sato, T. Sato, T. Kato, Y. Tachibana, “Discovery of S-217622, a Noncovalent Oral SARS-CoV-2 3CL Protease Inhibitor Clinical Candidate for Treating COVID-19” *J. Med. Chem.* **2022**, *65*, 6499–6512.

[7] B. K. Shoichet, “Virtual screening of chemical libraries.” *Nature* **2004**, *432*, 862–5.

[8] M. R. Berthold, N. Cebron, F. Dill, T. R. Gabriel, T. Meinl, P. Ohl, K. Thiel, B. Wiswedel, *KNIME-The Konstanz Information Miner Version 2.0 and Beyond*, **n.d.**

[9] Openeye, Cadence Molecular Sciences **2019**.

[10] C.-H. Zhang, E. A. Stone, M. Deshmukh, J. A. Ippolito, M. M. Ghahremanpour, J. Tirado-Rives, K. A. Spasov, S. Zhang, Y. Takeo, S. N. Kudalkar, Z. Liang, F. Isaacs, B. Lindenbach, S. J. Miller, K. S. Anderson, W. L. Jorgensen, “Potent Noncovalent Inhibitors of the Main Protease of SARS-CoV-2 from Molecular Sculpting of the Drug Perampanel Guided by Free Energy Perturbation Calculations” *ACS Cent. Sci.* **2021**, *7*, 467–475.

[11] Openeye, Cadence Molecular Sciences, “Color Features,” can be found under https://docs.eyesopen.com/applications/rocs/theory/shape_color.html, **2026**.

[12] Openeye, Cadence Molecular Sciences, “Shape Theory,” can be found under https://docs.eyesopen.com/applications/rocs/theory/shape_shape.html, **2026**.

[13] P. C. D. Hawkins, A. G. Skillman, A. Nicholls, “Comparison of shape-matching and docking as virtual screening tools.” *J. Med. Chem.* **2007**, DOI 10.1021/JM0603365.

[14] G. M. Sastry, M. Adzhigirey, T. Day, R. Annabhimoju, W. Sherman, “Protein and ligand preparation: parameters, protocols, and influence on virtual screening enrichments” *J. Comput. Aided Mol. Des.* **2013**, *27*, 221–234.

[15] Schrodinger **2019**.

[16] L. Schrödinger **2021**.

[17] *Schrödinger Release 2021-3 Maest. Schrödinger LLC N. Y. NY 2018* **2021**.

[18] *Schrödinger Release 2021-3 Epik Schrödinger LLC N. Y. NY 2021* **n.d.**

[19] J. C. Shelley, A. Cholleti, L. L. Frye, J. R. Greenwood, M. R. Timlin, M. Uchimaya, "Epik: A software program for pKa prediction and protonation state generation for drug-like molecules" *J. Comput. Aided Mol. Des.* **2007**, *21*, 681–691.
[20] R. A. Friesner, R. B. Murphy, M. P. Repasky, L. L. Frye, J. R. Greenwood, T. A. Halgren, P. C. Sanschagrin, D. T. Mainz, "Extra precision glide: Docking and scoring incorporating a model of hydrophobic enclosure for protein-ligand complexes" *J. Med. Chem.* **2006**, *49*, 6177–6196.
[21] M. Sándor, R. Kiss, G. M. Keserű, "Virtual Fragment Docking by Glide: a Validation Study on 190 Protein−Fragment Complexes" *J. Chem. Inf. Model.* **2010**, *50*, 1165–1172.
[22] R. A. Friesner, J. L. Banks, R. B. Murphy, T. A. Halgren, J. J. Klicic, D. T. Mainz, M. P. Repasky, E. H. Knoll, M. Shelley, J. K. Perry, D. E. Shaw, P. Francis, P. S. Shenkin, "Glide: A New Approach for Rapid, Accurate Docking and Scoring. 1. Method and Assessment of Docking Accuracy" *J. Med. Chem.* **2004**, *47*, 1739–1749.
[23] *Schrödinger Release 2021-3 Glide Schrödinger LLC N. Y. NY 2018* **2021**.
[24] S. Günther, P. Y. A. Reinke, Y. Fernández-García, J. Lieske, T. J. Lane, H. M. Ginn, F. H. M. Koua, C. Ehrt, W. Ewert, D. Oberthuer, O. Yefanov, S. Meier, K. Lorenzen, B. Krichel, J.-D. Kopicki, L. Gelisio, W. Brehm, I. Dunkel, B. Seychell, H. Gieseler, B. Norton-Baker, B. Escudero-Pérez, M. Domaracky, S. Saouane, A. Tolstikova, T. A. White, A. Hänle, M. Groessler, H. Fleckenstein, F. Trost, M. Galchenkova, Y. Gevorkov, C. Li, S. Awel, A. Peck, M. Barthelmess, F. Schlünzen, P. Lourdu Xavier, N. Werner, H. Andaleeb, N. Ullah, S. Falke, V. Srinivasan, B. A. França, M. Schwinzer, H. Brognaro, C. Rogers, D. Melo, J. J. Zaitseva-Doyle, J. Knoska, G. E. Peña-Murillo, A. R. Mashhour, V. Hennicke, P. Fischer, J. Hakanpää, J. Meyer, P. Gribbon, B. Ellinger, M. Kuzikov, M. Wolf, A. R. Beccari, G. Bourenkov, D. Von Stetten, G. Pompidor, I. Bento, S. Panneerselvam, I. Karpics, T. R. Schneider, M. M. Garcia-Alai, S. Niebling, C. Günther, C. Schmidt, R. Schubert, H. Han, J. Boger, D. C. F. Monteiro, L. Zhang, X. Sun, J. Pletzer-Zelgert, J. Wollenhaupt, C. G. Feiler, M. S. Weiss, E.-C. Schulz, P. Mehrabi, K. Karničar, A. Usenik, J. Loboda, H. Tidow, A. Chari, R. Hilgenfeld, C. Uetrecht, R. Cox, A. Zaliani, T. Beck, M. Rarey, S. Günther, D. Turk, W. Hinrichs, H. N. Chapman, A. R. Pearson, C. Betzel, A. Meents, "X-ray screening identifies active site and allosteric inhibitors of SARS-CoV-2 main protease" *Science* **2021**, *372*, 642–646.
[25] N. Drayman, J. K. DeMarco, K. A. Jones, S.-A. Azizi, H. M. Froggatt, K. Tan, N. I. Maltseva, S. Chen, V. Nicolaescu, S. Dvorkin, K. Furlong, R. S. Kathayat, M. R. Firpo, V. Mastrodomenico, E. A. Bruce, M. M. Schmidt, R. Jedrzejczak, M. Á. Muñoz-Alía, B. Schuster, V. Nair, K. Han, A. O'Brien, A. Tomatsidou, B. Meyer, M. Vignuzzi, D. Missiakas, J. W. Botten, C. B. Brooke, H. Lee, S. C. Baker, B. C. Mounce, N. S. Heaton, W. E. Severson, K. E. Palmer, B. C. Dickinson, A. Joachimiak, G. Randall, S. Tay, "Masitinib is a broad coronavirus 3CL inhibitor that blocks replication of SARS-CoV-2" *Science* **2021**, *373*, 931–936.
[26] C.-H. Zhang, E. A. Stone, M. Deshmukh, J. A. Ippolito, M. M. Ghahremanpour, J. Tirado-Rives, K. A. Spasov, S. Zhang, Y. Takeo, S. N. Kudalkar, Z. Liang, F. Isaacs, B. Lindenbach, S. J. Miller, K. S. Anderson, W. L. Jorgensen, "Potent Noncovalent Inhibitors of the Main Protease of SARS-CoV-2 from Molecular Sculpting of the Drug Perampanel Guided by Free Energy Perturbation Calculations" *ACS Cent. Sci.* **2021**, *7*, 467–475.
[27] A. Clyde, S. Galanie, D. W. Kneller, H. Ma, Y. Babuji, B. Blaiszik, A. Brace, T. Brettin, K. Chard, R. Chard, L. Coates, I. Foster, D. Hauner, V. Kertesz, N. Kumar, H. Lee, Z. Li, A. Merzky, J. G. Schmidt, L. Tan, M. Titov, A. Trifan, M. Turilli, H. Van Dam, S. C. Chennubhotla, S. Jha, A. Kovalevsky, A. Ramanathan, M. S. Head, R. Stevens, "High-Throughput Virtual Screening and Validation of a SARS-CoV-2 Main Protease Noncovalent Inhibitor" *J. Chem. Inf. Model.* **2022**, *62*, 116–128.
[28] M. Jacobsson, P. Lidén, E. Stjernschantz, H. Boström, U. Norinder, "Improving structure-based virtual screening by multivariate analysis of scoring data." *J. Med. Chem.* **2003**, *46*, 5781–9.
[29] E. A. Hecker, C. Duraiswami, T. A. Andrea, D. J. Diller, "Use of Catalyst Pharmacophore Models for Screening of Large Combinatorial Libraries" *J. Chem. Inf. Comput. Sci.* **2002**, *42*, 1204–1211.
[30] T. Fawcett, "An introduction to ROC analysis" *Pattern Recognit. Lett.* **2006**, *27*, 861–874.
[31] R. C. Braga, C. H. Andrade, "Assessing the Performance of 3D Pharmacophore Models in Virtual Screening: How Good are They?" *Curr. Top. Med. Chem.* **2013**, *13*, 1127–1138.
[32] S. Genheden, U. Ryde, "The MM/PBSA and MM/GBSA methods to estimate ligand-binding affinities" *Expert Opin. Drug Discov.* **2015**, *10*, 449–461.
[33] D. Rogers, M. Hahn, "Extended-connectivity fingerprints" *J. Chem. Inf. Model.* **2010**, *50*, 742–754.
[34] S. Riniker, G. A. Landrum, "Open-source platform to benchmark fingerprints for ligand-based virtual screening" *J. Cheminformatics* **2013**, *5*, 26.
[35] G. Landrum **2017**.
[36] S. Anderson, "Graphical representation of molecules and substructure-search queries in MACCS" *J. Mol. Graph.* **1984**, *2*, 83–90.
[37] P. S. Foundation **2020**.
[38] A. Cherkasov, E. N. Muratov, D. Fourches, A. Varnek, I. I. Baskin, M. Cronin, J. Dearden, P. Gramatica, Y. C. Martin, R. Todeschini, V. Consonni, V. E. Kuz'min, R. Cramer, R. Benigni, C. Yang, J. Rathman, L. Terfloth, J. Gasteiger, A. Richard, A. Tropsha, "QSAR Modeling: Where Have You Been? Where Are You Going To?" *J. Med. Chem.* **2014**, *57*, 4977–5010.
[39] T. Hastie, R. Tibshirani, J. Friedman in *Elem. Stat. Learn. Data Min. Inference Predition*, Springer, **2009**.

[40] C. Cortes, V. Vapnik, "Support-Vector Networks" *Mach. Learn.* **1995**, *20*, 273–297.
[41] L. E. O. Breiman, "Random Forests." *Mach. Learn.* **2001**, *45*, 5–32.
[42] G. Ke, Q. Meng, T. Finley, T. Wang, W. Chen, W. Ma, Q. Ye, T. Y. Liu, "LightGBM: A highly efficient gradient boosting decision tree" *Adv. Neural Inf. Process. Syst.* **2017**, *2017-Decem*, 3147–3155.
[43] F. Pedregosa, G. Varoquaux, A. Gramfort, V. Michel, B. Thirion, O. Grisel, M. Blondel, P. Prettenhofer, R. Weiss, V. Dubourg, J. Vanderplas, A. Passos, D. Cournapeau, M. Brucher, M. Perrot, É. Duchesnay, "Scikit-learn: Machine learning in Python" *J. Mach. Learn. Res.* **2011**.
[44] T. Head, M. Kumar, H. Nahrstaedt, G. Louppe, I. Shcherbatyi **2020**, DOI 10.5281/zenodo.4014775.
[45] J. Wu, X. Y. Chen, H. Zhang, L. D. Xiong, H. Lei, S. H. Deng, "Hyperparameter optimization for machine learning models based on Bayesian optimization" *J. Electron. Sci. Technol.* **2019**, *17*, 26–40.
[46] D. Gadaleta, G. F. Mangiatordi, M. Catto, A. Carotti, O. Nicolotti, "Applicability Domain for QSAR Models" *Int. J. Quant. Struct.-Prop. Relatsh.* **2016**, *1*, 45–63.
[47] O. S. Software **2019**.
[48] O. S. Software **2019**.
[49] P. C. D. Hawkins, A. G. Skillman, G. L. Warren, B. A. Ellingson, M. T. Stahl, "Conformer generation with OMEGA: Algorithm and validation using high quality structures from the protein databank and cambridge structural database" *J. Chem. Inf. Model.* **2010**, *50*, 572–584.
[50] C. L. Manach, D. G. Cabrera, F. Douelle, A. T. Nchinda, Y. Younis, D. Taylor, L. Wiesner, K. L. White, E. Ryan, C. March, S. Duffy, V. M. Avery, D. Waterson, M. J. Witty, S. Wittlin, S. A. Charman, L. J. Street, K. Chibale, "Medicinal chemistry optimization of antiplasmodial imidazopyridazine hits from high throughput screening of a SoftFocus kinase library: Part 1" *J. Med. Chem.* **2014**, *57*, DOI 10.1021/jm500098s.
[51] M. Leshabane, G. A. Dziwornu, D. Coertzen, J. Reader, P. Moyo, M. V. D. Watt, K. Chisanga, C. Nsanzubuhoro, R. Ferger, E. Erlank, N. Venter, L. Koekemoer, K. Chibale, L. M. Birkholtz, "Benzimidazole Derivatives Are Potent against Multiple Life Cycle Stages of Plasmodium falciparum Malaria Parasites" *ACS Infect. Dis.* **2021**, *7*, DOI 10.1021/acsinfecdis.0c00910.
[52] C. L. Manach, J. Dam, J. G. Woodland, G. Kaur, L. P. Khonde, C. Brunschwig, M. Njoroge, K. J. Wicht, A. Horatscheck, T. Paquet, G. A. Boyle, L. Gibhard, D. Taylor, N. Lawrence, T. Yeo, S. Mok, R. T. Eastman, D. Dorjsuren, D. C. Talley, H. Guo, A. Simeonov, J. Reader, M. V. D. Watt, E. Erlank, N. Venter, J. W. Zawada, A. Aswat, L. Nardini, T. L. Coetzer, S. B. Lauterbach, B. C. Bezuidenhout, A. Theron, D. Mancama, L. L. Koekemoer, L. M. Birkholtz, S. Wittlin, M. Delves, S. Ottilie, E. A. Winzeler, D. Smith, D. A. Fidock, L. J. Street, G. S. Basarab, J. Duffy, K. Chibale, "Identification and Profiling of a Novel Diazaspiro[3.4]octane Chemical Series Active against Multiple Stages of the Human Malaria Parasite Plasmodium falciparum and Optimization Efforts" *J. Med. Chem.* **2021**, *64*, DOI 10.1021/acs.jmedchem.1c00034.
[53] G. A. Dziwornu, D. Coertzen, M. Leshabane, C. M. Korkor, C. K. Cloete, M. Njoroge, L. Gibhard, N. Lawrence, J. Reader, M. V. D. Watt, S. Wittlin, L. M. Birkholtz, K. Chibale, "Antimalarial Benzimidazole Derivatives Incorporating Phenolic Mannich Base Side Chains Inhibit Microtubule and Hemozoin Formation: Structure-Activity Relationship and in Vivo Oral Efficacy Studies" *J. Med. Chem.* **2021**, *64*, DOI 10.1021/acs.jmedchem.1c00354.
[54] H. Alalam, S. Sigurdardóttir, C. Bourgard, I. Tiukova, R. D. King, M. Grøtli, P. Sunnerhagen, "A Genetic Trap in Yeast for Inhibitors of SARS-CoV-2 Main Protease" *mSystems* **2021**, *6*, DOI 10.1128/mSystems.01087-21.
[55] S. Sigurdardóttir, S. F. Silva, I. Tiukova, H. Alalam, R. D. King, M. Grøtli, L. A. Eriksson, P. Sunnerhagen, "An automated positive selection screen in yeast provides support for boron-containing compounds as inhibitors of SARS-CoV-2 main protease" *Microbiol. Spectr.* **2024**, DOI 10.1128/spectrum.01249-24.
